# *STXBP1* Syndrome Is Characterized by Inhibition-Dominated Dynamics of Resting-State EEG

**DOI:** 10.3389/fphys.2021.775172

**Published:** 2021-12-23

**Authors:** Simon J. Houtman, Hanna C. A. Lammertse, Annemiek A. van Berkel, Ganna Balagura, Elena Gardella, Jennifer R. Ramautar, Chiara Reale, Rikke S. Møller, Federico Zara, Pasquale Striano, Mala Misra-Isrie, Mieke M. van Haelst, Marc Engelen, Titia L. van Zuijen, Huibert D. Mansvelder, Matthijs Verhage, Hilgo Bruining, Klaus Linkenkaer-Hansen

**Affiliations:** ^1^Department of Integrative Neurophysiology, Center for Neurogenomics and Cognitive Research, (CNCR), Amsterdam Neuroscience, VU University Amsterdam, Amsterdam, Netherlands; ^2^Department of Functional Genomics, Center for Neurogenomics and Cognitive Research, (CNCR), Amsterdam Neuroscience, VU University Amsterdam, Amsterdam, Netherlands; ^3^Department of Human Genetics, Amsterdam UMC, Amsterdam, Netherlands; ^4^IRCCS Istituto Giannina Gaslini, Genova, Italy; ^5^Department of Neurosciences, Rehabilitation, Ophthalmology, Genetics, Maternal and Child Health, University of Genova, Genova, Italy; ^6^Department of Epilepsy Genetics and Personalized Treatment, Danish Epilepsy Centre, Dianalund, Denmark; ^7^Department of Regional Health Research, University of Southern Denmark, Odense, Denmark; ^8^ Member of the ERN EpiCARE; ^9^Child and Adolescent Psychiatry and Psychosocial Care, Emma Children’s Hospital, Amsterdam UMC, Vrije Universiteit Amsterdam, Amsterdam, Netherlands; ^10^Department of Clinical and Experimental Medicine, Epilepsy Center, University Hospital of Messina, Messina, Italy; ^11^Department of Pediatric Neurology, Amsterdam UMC, University of Amsterdam, Amsterdam, Netherlands; ^12^Research Institute of Child Development and Education, University of Amsterdam, Amsterdam, Netherlands; ^13^N=You Neurodevelopmental Precision Center, Amsterdam Neuroscience, Amsterdam Reproduction and Development, Amsterdam UMC, Amsterdam, Netherlands; ^14^Levvel, Center for Child and Adolescent Psychiatry, Amsterdam, Netherlands

**Keywords:** MUNC18-1, SNAREopathies, EEG, excitation-inhibition balance, *fE/I*, aperiodic exponent

## Abstract

*STXBP1* syndrome is a rare neurodevelopmental disorder caused by heterozygous variants in the *STXBP1* gene and is characterized by psychomotor delay, early-onset developmental delay, and epileptic encephalopathy. Pathogenic *STXBP1* variants are thought to alter excitation-inhibition (E/I) balance at the synaptic level, which could impact neuronal network dynamics; however, this has not been investigated yet. Here, we present the first EEG study of patients with *STXBP1* syndrome to quantify the impact of the synaptic E/I dysregulation on ongoing brain activity. We used high-frequency-resolution analyses of classical and recently developed methods known to be sensitive to E/I balance. EEG was recorded during eyes-open rest in children with *STXBP1* syndrome (*n* = 14) and age-matched typically developing children (*n* = 50). Brain-wide abnormalities were observed in each of the four resting-state measures assessed here: (i) slowing of activity and increased low-frequency power in the range 1.75–4.63 Hz, (ii) increased long-range temporal correlations in the 11–18 Hz range, (iii) a decrease of our recently introduced measure of functional E/I ratio in a similar frequency range (12–24 Hz), and (iv) a larger exponent of the 1/f-like aperiodic component of the power spectrum. Overall, these findings indicate that large-scale brain activity in *STXBP1* syndrome exhibits inhibition-dominated dynamics, which may be compensatory to counteract local circuitry imbalances expected to shift E/I balance toward excitation, as observed in preclinical models. We argue that quantitative EEG investigations in *STXBP1* and other neurodevelopmental disorders are a crucial step to understand large-scale functional consequences of synaptic E/I perturbations.

## Introduction

*STXBP1* syndrome is an early-onset neurodevelopmental disorder caused by variants in the *STXBP1* gene (Saitsu et al., 2008; Stamberger et al., 2016). *STXBP1* encodes the protein MUNC18-1, a key organizer protein for the SNARE-complex (Südhof and Rothman, 2009), which is essential for synaptic transmission (Verhage et al., 2000). The most prominent feature of SNAREopathies, and *STXBP1* variants specifically, is a degree of psychomotor retardation and intellectual disability (Stamberger et al., 2016; Abramov et al., 2021). The clinical phenotype is further characterized by epilepsy, EEG abnormalities, and a variety of neuropsychiatric symptoms (Deprez et al., 2010; Saitsu et al., 2010; Mastrangelo et al., 2013; Stamberger et al., 2016; Abramov et al., 2021).

Multiple lines of evidence have demonstrated that pathogenic *STXBP1* variants cause haploinsufficiency (Saitsu et al., 2010; Guiberson et al., 2018; Kovačević et al., 2018). Heterozygous *Stxbp1* null mouse models recapitulate clinical features such as cognitive impairment, behavioral symptoms, seizures, and abnormal EEG (Hager et al., 2014; Miyamoto et al., 2017, 2019; Kovačević et al., 2018; Orock et al., 2018; Chen et al., 2020). Excitatory and inhibitory neurotransmission are differentially affected by *STXBP1* haploinsufficiency, with inhibitory transmission being more profoundly affected in both single neurons and cortico-cortical circuitry (Toonen et al., 2006; Chen et al., 2020), whereas reduced excitatory signaling was observed in cortico-striatal projections (Miyamoto et al., 2019). Thus, alterations in E/I balance are observed in local brain circuits, which may lead to E/I imbalances at the network level and contribute to clinical symptomatology. A growing body of evidence suggests that E/I imbalances at the synapse play a key role in the pathogenesis of neurodevelopmental disorders (Rubenstein and Merzenich, 2003; Ramamoorthi and Lin, 2011; Nelson and Valakh, 2015; Foss-Feig et al., 2017; Ghatak et al., 2021) and have a strong impact on neuronal network performance, cognition, and behavior (Rubenstein and Merzenich, 2003). However, the interrelations between cellular deficits observed in animal models, possible network-level disturbances, and clinical symptoms remain unelucidated. To bridge this gap, quantitative EEG analyses allow to assess network-level alterations in patients with *STXBP1* syndrome compared to control individuals.

Hence, the aim of this study was to investigate whether *STXBP1* haploinsufficiency at the synapse shifts E/I balance of network-level brain dynamics. To this end, we aggregated EEG recordings of patients with *STXBP1* syndrome (*n* = 14) and typically developing children (TDC; *n* = 50). Three E/I-sensitive measures were assessed: the 1/f exponent of the power spectrum (Gao et al., 2017; Donoghue et al., 2020), long-range temporal correlations of oscillations (Linkenkaer-Hansen et al., 2001; Hardstone et al., 2012), and the newly developed measure of functional E/I ratio (*fE/I*; Bruining et al., 2020). These analyses reveal pronounced E/I disturbances in the patients with *STXBP1* syndrome toward a more inhibition-dominated state.

## Materials and Methods

### Participants and Study Design

This study is a collaborative multi-center case-control study with participants recorded at five institutes. EEG was recorded prospectively for this study at Giannina Gaslini Institute in Genova, Italy (*n* = 3) and Vrije Universiteit Amsterdam, Netherlands (*n* = 5). A subset of recordings was collected retrospectively from the Danish Epilepsy Centre in Dianalund, Denmark (*n* = 6). Recordings from TDC, i.e., children who achieved age-appropriate developmental milestones, were collected retrospectively from UMC Utrecht (*n* = 29) and University of Amsterdam (*n* = 21). Data collection was approved by the Medical Ethics Review Committees associated with the specific institutes and following the Declaration of Helsinki and relevant guidelines and regulations. Written informed consent was received from participants or (one of) their legal guardians prior to prospectively performed measurements.

#### Children With *STXBP1* Syndrome: Amsterdam Sample

Five children (age 3.3–10.5 years, 2 females) were recorded prospectively at Vrije Universiteit Amsterdam and Amsterdam University Medical Center in Amsterdam, Netherlands. Data were collected during two separate STXBP1-clinic days organized to exchange information on *STXBP1* syndrome between researchers, clinicians, parents, and children. EEG recordings were conducted during 5–14 min eyes-open rest using a NetAmps300 amplifier (Electrical Geodesics Incorporated) and a 129-channel HydroCel Geodesic sensor net. Sampling rate was 1,000 Hz, reference electrode Cz.

#### Children With *STXBP1* Syndrome: Denmark Sample

Six children (age 8–14 years, 4 females) were measured in a clinical setting at the Filadelfia Epilepsy Centre in Dianalund, Denmark, and collected retrospectively. EEG recordings were conducted during 1–8 min eyes-open rest using a NicoletOne nEEG v5.95.0.25 (Natus Medical Incorporated) setup with 19 electrodes placed according to the 10–20 system. Sampling rate varied across recordings between 200 and 2,000 Hz, reference electrode Cz.

#### Children With *STXBP1* Syndrome: Italy Sample

Three children (age 2.8–8.3 years, 0 female) were recorded in a clinical setting at the Giannina Gaslini Institute (IRCCS Research Hospital) in Genova, Italy, and collected for this study prospectively. EEG was recorded during 6.5–19 min eyes-open rest using 19 electrodes placed according to the 10–20 system. Sampling rate was 500 Hz, reference electrode Cz.

#### Typically Developing Children: Utrecht Sample

EEG recordings of 29 TDC (age 7–16 years, 14 females) were conducted at the developmental disorder unit at UMC Utrecht as part of two ongoing studies with identical measurement protocols: Sensory information Processing in Autism and Childhood Epilepsy (SPACE) and Bumetanide in Autism Medication and Biomarker (BAMBI; Bruining et al., 2020). The control sample was recruited from children attending non-special education. Exclusion criteria were a history of behavioral or learning problems, a diagnosis of any neurodevelopmental condition, or any other major health issue. EEG recordings were conducted during 3–5 min eyes-open rest using a 64-channel BioSemi setup at a sampling rate of 2,048 Hz and Driven Right Leg passive electrode.

#### Typically Developing Children: University of Amsterdam Sample

EEG recordings of 21 TDC (8 children at age 35 months, 13 children at age 47 months) were conducted at University of Amsterdam as part of the NWO-funded Dutch Dyslexia Programme. Children included here were part of the control group of a study on preliteracy signatures related to poor-reading abilities in resting-state EEG (Schiavone et al., 2014). EEG recordings were conducted during 3–5 min eyes-open rest using a Neuroscan setup with a SynAmps2 64-channel net. Channels were placed according to the 10–20 international system. Sampling rate was 500 Hz, mastoids were used as references.

### Demographics and Clinical Scales

For children with *STXBP1* syndrome, we collected the following demographics and clinical information: age, gender, mutation type (i.e., deletion, missense or frameshift, splice-site, and stop or intragenic), specific mutation site, whether or not the variant was *de novo*, epilepsy diagnosis, medication at the time of recording, developmental delay, and whether or not motor abilities were affected. Clinical severity was measured using three different scales: Gross Motor Function Classification System (GMFCS), Manual Ability Classification System (MACS), and Communication Function Classification System (CFCS). GMFCS quantifies the ability to perform common movements such as sitting, walking, and use of mobility devices (Palisano et al., 1997). MACS measures how children use their hands to handle objects in daily life (Eliasson et al., 2017). CFCS quantifies the effectiveness of everyday communication (Hidecker et al., 2011). These scales consist of a five-point Likert scale, 1 indicating low and 5 reflecting high clinical severity. Visualization of the crystal structure and mutation sites was performed using UCSF Chimera software (v. 1.14 developed by the Resource for Biocomputing, Visualization, and Informatics at the University of California, San Francisco, with support from NIH P41-GM103311; Pettersen et al., 2004).

### Preprocessing

**Figure 1:**
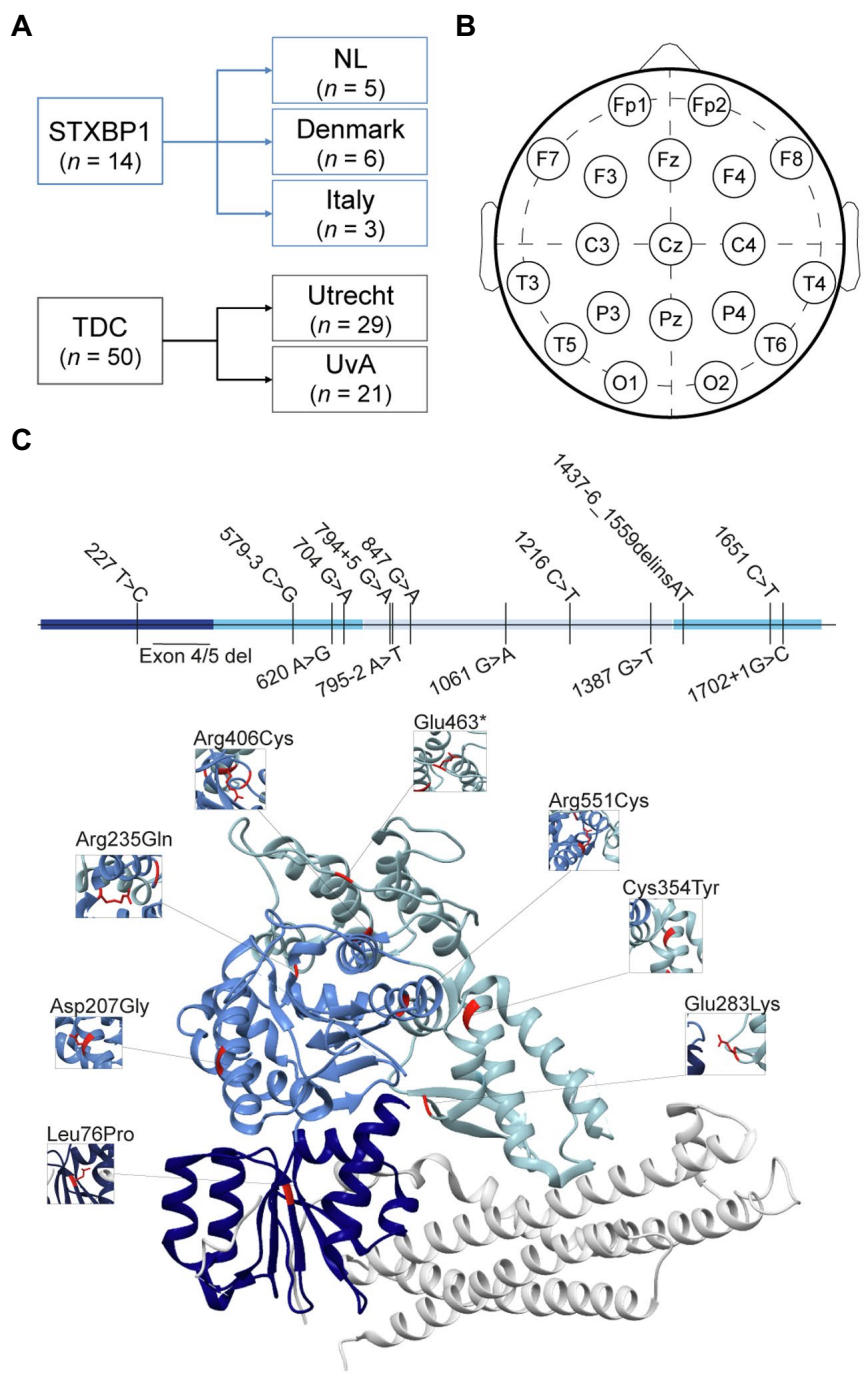
Demographics, electrode positioning and mutation details. **(A)** Recordings of *STXBP1* syndrome patients (*n* = 14) were collected from three recording sites. Recordings from Denmark were collected retrospectively, whereas recordings from Italy and the Netherlands were performed prospectively for this study. Typically developing children (TDC, *n* = 50), were collected from SPACE/BAMBI programs from UMC Utrecht (*n* = 29) and University of Amsterdam (*n* = 21). **(B)** Electrode map for selected 19 channels of 10–20 system. **(C)** Genetic mutations of *STXBP1* syndrome patients indicated in the primary sequence (top) and crystal structure of the STXBP1/Munc18-1-syntaxin1 dimer (Misura et al., 2000; bottom). The three domains in the MUNC18-1 protein are indicated in three shades of blue. The white protein structure is the STXBP1/Munc18-1 binding partner Syntaxin1. In the primary sequence, the mutations are indicated as the nucleotide numbers (starting at 1 at the ATG start site) and the alterations in nucleotides. Inserted nucleotides (causing frame shift) are indicated with a plus symbol, intronic positions are indicated as the position relative to the exon, using a minus or plus sign, deletions are indicated with “del”; “delins” indicates a combination of deleted nucleotides followed by (partial) insertion of others. In the crystal structure, missense mutations are indicated in the structure in red with the amino acid number and change.

EEG analyses were done in MATLAB R2020a, version 9.8 (The Mathworks, Inc., Natick, MA). Preprocessing was done using EEGLAB v2020.0 (Delorme and Makeig, 2004) and additional custom-made scripts developed for this study (Supplementary Figure 1). Continuous EEG recordings were bandpass-filtered for 1–45 Hz using a FIR-filter with a Hamming window. Bad channels were defined as electrodes with excessive line noise or flat signal due to low conductance with the scalp and were detected automatically using a joint probability, power spectrum, or kurtosis exceeding three standard deviations from all electrodes. Bad channels were interpolated with a spherical spline using all channels in the original dataset. Recordings were re-referenced to the average of all electrodes. To aggregate and compare recordings across institutes, 19 channels of the 10–20 system available across all setups were selected: Fp1, Fp2, F3, F4, C3, C4, P3, P4, O1, O2, F7, F8, T3, T4, T5, T6, Fz, Cz, Pz (Figure 1B). Recordings were not down-sampled to a common sampling rate. However, we also report the results of analyses performed at a common sampling rate of 200 Hz (Supplementary Figure 4).

To remove transient artifacts, recordings were segmented into one-second data epochs. Epochs were removed if the amplitude exceeded [−150, 150] μV. For three *STXBP1* recordings, amplitude range was set to [−500, 500] μV due to a larger amplitude range without the presence of artifacts, to prevent excessive loss of clean EEG segments. Eye artifacts were removed using independent component analysis (ICA), which unmixes EEG channel data into components that are statistically independent of each other (Bell and Sejnowski, 1995). ICA can be used to separate distinct physiological and non-physiological sources of EEG data and works particularly well for separating eye artifacts (Vigário, 1997). Here, the rank of the EEG channel data was used for dimensionality reduction using principal component analysis before running ICA. Components with clear horizontal eye movements (i.e., HEOG) or vertical eye movements (i.e., VEOG) were rejected using *ICLabel*, which is an automatic IC classifier that contains spatiotemporal measures for over 200,000 ICs from more than 6,000 EEG recordings labeled through crowdsourcing (Pion-Tonachini et al., 2019). Removed components were visually inspected and only included strong horizontal and vertical eye components. After artifact removal, epochs were inspected in continuous windows of 10 s by two EEG experts to remove noisy epochs that were not picked up by the automatic procedure but could bias estimation of EEG measures. Epochs were judged as *good* or *bad*. The proportionate agreement between the two experts was 97.2%. Epochs with a discrepancy between raters and epochs that were judged as *bad* by both raters were removed.

On average, 13.7% of electrodes in the original dataset were detected as noisy and interpolated (Mn_TDC_ = 13.6% ± 2.7%, Mn_STXBP1_ = 14.1% ± 5.3%, *t*(62) = 0.55, *p* = 0.59; Supplementary Figure 2A). Out of the 19 channels that were selected, 10.8% were interpolated in the original dataset (Mn_TDC_ = 9.8% ± 5.0%, Mn_STXBP1_ = 14.3% ± 5.6%, *t*(62) = 2.9, *p* = 0.0052). For each electrode, the average percentage of recordings was computed for which that electrode was detected as noisy and interpolated. Bad electrodes were mainly at the rim of the head (Supplementary Figure 2A, topographical inset). The mean percentage of noisy epochs was 9.53% (Mn_TDC_ = 7.4% ± 9.9%, Mn_STXBP1_ = 17.1% ± 16.6%, *t*(62) = 2.8, *p* = 0.0075; Supplementary Figure 2B). The mean number of independent components removed was 2 (Mn_TDC_ = 2.1 ± 0.9, Mn_STXBP1_ = 2.3 ± 1.2, *t*(62) = 0.58, *p* = 0.57; Supplementary Figure 2C). Mean signal length after preprocessing was 265 s (Mn_TDC_ = 232 s ± 69 s, Mn_STXBP1_ = 383 s ± 234 s, *t*(62) = 11.8, *p* = 1.9e-17; Supplementary Figure 2D). Typical horizontal and vertical eye component selected by *ICLabel* (Supplementary Figure 2E). To test whether preprocessing influenced our results, analysis of covariance (ANCOVA) was performed with the value of each EEG measure as the dependent variable, group as independent factor and cleaning statistic as continuous covariate. Cleaning statistics tested were percentage of bad channels (out of the 19 selected 10–20 channels), percentage of bad epochs, number of removed independent components, and signal length after preprocessing. Significance was defined as *p* < 0.05, Bonferroni-corrected for the number of EEG measures times cleaning statistics (i.e., $p<\frac{.05}{16}$). *F*-values of factor group of the ANCOVA were visualized on a heatmap with EEG measures on the rows and covariates on the columns (Supplementary Figure 2F). Group (i.e., TDC versus *STXBP1* syndrome) was significant for all EEG measures tested here when including each cleaning statistic as a covariate (Supplementary Figure 2F). *F*-values of the different covariates were visualized in a similar fashion (Supplementary Figure 2G). Number of removed independent components had a significant effect on DFA [*F*(1,60) = 4.5, *p* = 0.04], however, not after Bonferroni correction. There was a significant effect of percentage of bad epochs on *fE/I* [*F*(1,60) = 5.3, *p* = 0.03] but not after Bonferroni correction. Number of removed independent components had a significant effect on *fE/I* after Bonferroni correction [*F*(1,60) = 7.3, *p* = 0.009]. Percentage of bad epochs had a significant effect on aperiodic exponent after Bonferroni correction [*F*(1,60) = 12.7, *p* = 0.0007].

### Computation of EEG Measures

#### Spectral Power

Power spectral density was computed for all channels using *Welch*’s method (2-s Hamming windows, 50% overlap). The length of the FFT was determined as *fs*/0.125 – where *fs* is the sampling frequency – to obtain a frequency resolution of 0.125 Hz for all recordings.

#### Long-Range Temporal Correlations

To investigate whether temporal structure of brain oscillations was altered in children with *STXBP1* syndrome compared with TDC, long-range temporal correlations in the amplitude modulation of oscillations were calculated using detrended fluctuation analysis (DFA; Peng et al., 1995; Linkenkaer-Hansen et al., 2001; Hardstone et al., 2012). EEG signals were filtered in frequency bins of 1 Hz, using a Hamming-windowed FIR-filter with a transition bandwidth of 1 Hz. Amplitude envelope was obtained by taking the absolute of the Hilbert transform and demeaned to yield a time series of fluctuations around the mean. The demeaned amplitude envelope time series was split into 50% overlapping windows, the cumulative sum of the envelope calculated, and the linear trend removed from each window. The fluctuation of the resulting signal was computed as the standard deviation (SD) per window and the average fluctuation was derived by computing the mean SD across windows. This process was repeated for windows of different logarithmically-spaced time-scales between 2 and 20 s for frequencies above 8 Hz and 4–20 s for frequencies below 8 Hz. Smaller time-scales were not considered to avoid biasing the scaling-law estimation from temporal correlations induced by the FIR-filter (Hardstone et al., 2012). The DFA exponent is then defined as the exponent of the least-squares linear fit of mean fluctuation over time-scale on a log-log scale. DFA of 0.5 indicates no autocorrelations; DFA between 0.5 and 1 indicates positive autocorrelations and reflects the presence of LRTC.

#### Excitation/Inhibition Ratio (*fE/I*)

Network excitation-inhibition ratio was quantified using *fE/I*, which is an algorithm derived from the Critical Oscillations (CROS) model of ongoing neuronal activity (Poil et al., 2012; Bruining et al., 2020). In the CROS model, strong associations have been observed between structural E/I ratio, amplitude, and the DFA exponent of the amplitude modulation of oscillations. From these observations, it followed that *fE/I* can be estimated from the covariance of amplitude and a fluctuation function that correlates strongly with the DFA exponents. *fE/I* can be obtained from windows of merely a few seconds, which has been described in full detail in the reference paper (Bruining et al., 2020). Here, instead of computing *fE/I* for traditional EEG frequency bands, the signal of each electrode was filtered in frequency bins of 1 Hz using a Hamming-windowed FIR-filter with a transition bandwidth of 1 Hz, and *fE/I* was calculated for windows of 5 s with 80% overlap. *fE/I* < 1 indicates inhibition-dominated networks, *fE/I* > 1 reflects excitation-dominated networks and E/I-balanced networks will have *fE/I* ≈ 1.

#### Aperiodic Exponent

The power spectrum of neural data consists of a 1/f-like aperiodic component (Freeman and Zhai, 2009; He, 2014) as well as oscillatory components indicated by peaks rising above the aperiodic component (Donoghue et al., 2020). The aperiodic component has a $1/f^{\beta }$ decay of power with frequency, where β is the aperiodic exponent that determines the slope of the power spectrum. Recently, the aperiodic exponent has been associated with E/I ratio *in silico*, with smaller exponents being associated with higher E/I ratio (Gao et al., 2017). Here, we used the *FOOOF* algorithm (version 1.0.0) to compute the aperiodic exponent as an index of E/I balance (Donoghue et al., 2020). Power spectra were fit between 1 and 30 Hz using the settings (peak_width_limits = [1,6], max_n_peaks = 6, min_peak_height = 0.05, peak_threshold = 1.5, aperiodic_mode = “fixed”). Average $R^{2}$ of spectral fits was 0.95, indicating good fits.

### Statistical Analysis

Since *STXBP1* syndrome is a developmental disorder and because age was heterogeneously distributed within both patients and controls, comparisons between TDC and *STXBP1* syndrome were done while controlling for age. ANCOVA was used to compare between TDC and *STXBP1* syndrome. The value of each EEG measure was used as the dependent variable, group as an independent factor and age as a covariate. Recording site was not included as a covariate because of perfect multicollinearity with factor group and because of the low number of observations per level. Statistical significance was defined as *p* < 0.05. For power spectral density, DFA and *fE/I* computed across frequencies, ANCOVA was performed for individual frequency bins in the range 1–45 Hz. The width of frequency bins was 0.125 Hz for power (i.e., 353 bins) and 1 Hz for DFA and *fE/I* (i.e., 44 bins). Bonferroni correction was used to correct for multiple comparisons across frequency bins. For spatial analyses, statistical analyses were conducted at the single-electrode level. Bonferroni correction was used to correct for multiple comparisons across 19 electrodes, per topography. The *F*-value and *p*-value were reported for each ANCOVA. A partial correlation was used to test for associations between clinical scales and EEG measures while controlling for the effect of age. Linear regression was used to test whether EEG measures changed with age in TDC or *STXBP1* syndrome separately. The slope of the regression line was reported for each metric, per group. On topographies, electrode values depict the mean across subjects. Open white circles reflect significance at *p* < 0.05, whereas closed white circles indicate significance after Bonferroni correction.

### Data Availability Statement

Matlab code for computing high-frequency-resolution EEG measures and scripts to produce all figures are available at https://figshare.com/account/home#/projects/127670.

## Results

EEG recordings from 14 individuals with *STXBP1* syndrome were compared to those of 50 typically developing children (TDC; Figure 1A). To aggregate data from different recording sites, 19 channels common to all EEG setups were selected (Figure 1B). Recordings were rigorously preprocessed by two EEG experts using a semi-automatic procedure (Supplementary Figures 1, 2). The *STXBP1* syndrome patient group comprised different mutation type carriers, including missense, truncations, frameshift, intronic variants, and partial deletions (Figure 1C, top). Moreover, missense variants were located across the protein (Figure 1C, bottom). Of the 14 patients with *STXBP1* syndrome, 10 had been diagnosed with epilepsy of whom seven were using anti-epileptic medication(s) at the time of the EEG recording. The other patients with *STXBP1* syndrome had no history of seizures. All patients showed developmental delay and motor impairment, with varying degree of severity (Table 1). Thus, the *STXBP1* patient group in this study is representative of the genetic heterogeneity and the variability in the clinical presentation as previously reported (Stamberger et al., 2016; Abramov et al., 2021).

**Table 1:** Patient demographics and clinical scores.

#	Site	Sex	Mutation site	Age	Dev. delay	Epilepsy (Seizure type)	Current medication	Neurological features	Motor development	Language development	Neuropsychiatric features	GMFCS	MACS	CFCS
1	IT	m	c. 1702 + 1 G > C	2 years 10 months	y	y (tonic, myoclonic, and monthly clusters)	None	Axial and distal hypotonia; tremor	Some steps with assistance	No	Autistic features; higher pain threshold	IV	IV	IV
2	IT	m	Deletion exon 4&5	5 years 6 months	y	y (focal, tonic, and no clear triggers)	TPM, PB, and CBD oil. CLB when needed	Hypotonia, dystonia; buccal dyspraxia, hyporeflexia	Not able to walk	No	Autistic features; lower pain threshold	V	V	V
3	IT	m	c. 704 G > A p. Arg235Gln	8 years 3 months	y	y (seizure-free since 6 months)	None	Mild tremor, ataxia	Able to walk	No	Autistic features, bruxism	II	IV	IV
4	NL	m	c. 579–3 C > G	10 years 6 months	y	n	n/a	Hypotonia; tremor after waking up			Stereotypies; very active	II	III	IV
5	NL	m	c. 620 A > G p. Asp207Gly	6 years 3 months	y	n	n/a	Tremor	Able to walk		Diagnosed ASD; easily distracted	I	I	II
6	NL	m	c. 1216 C > T p. Arg406Cys	3 years 5 months	y	n	n/a	Hypotonia, ataxia, balance problems. Babinski sign, dysmetria	Unable to walk,	No		IV	n/a	IV
7	NL	m	c. 847 G > A p. Glu283Lys	7 years 6 months	y	y (tonic–clonic, focal, and tonic)	LTG	Dyspraxia, very moveable; tremor, ataxia	Able to walk with support,	Able to speak (correct grammar)	Autistic features, highly (sensory) sensitive; restless; loses concentration rapidly; regression after seizure clusters	I	II	III
8	NL	m	c. 227 T > C p. Leu76Pro	3 years 4 months	y	n	n/a	Babinski sign, able to grab objects,			Autistic features	IV	n/a	V
9	DK	f	c.1651C > T p. Arg551Cys	14 years	y	y (spasms, FIAS, tonic, and tonic–clonic)	CBZ, LEV, and VNS	Axial hypotonus, hypertonus	Unable to walk	No	Profound ID	V	V	V
10	DK	f	c.794 + 5G > A	8 years	y	y (FIAS, tonic, and tonic–clonic)	RFM, LTG, CLB, and VNS	Ataxia, poor coordination, hypotonia, oral dyspraxia	Able to walk	No	Autistic features	II	IV	III
11	DK	f	c.1387G > T p.Glu463*	14 years	y	y	PER, VPA, CLB, and VNS	n/a	Able to walk			n/a	n/a	n/a
12	DK	m	c.1437-6_1559delinsAT	10 years	y	y (spasms, FIAS, clonic, tonic-myoclonic, tonic–clonic, and SE)	CLB, LAC, and VNS	Spastic tetraparesis, hypotonia, dyskinesia	Unable to walk	No	Severe/profound ID	V	V	V
13	DK	m	c.795-2A > T	11 years	y	y (tonic and atonic)	PER, LAC, CLB, and RFM	Ataxia, poor coordination, dystonia, hypertonus	Able to walk	No	Autistic features, moderate–severe ID	II	IV	III
14	DK	f	c.1061G > A p. Cys354Tyr	13 years	y	y (currently seizure-free)	None	n/a	n/a	n/a	n/a	n/a	n/a	n/a

IT: Italy. NL: The Netherlands. DK: Denmark. Dev. delay: Developmental delay. GMFCS: Gross Motor Function Classification System. MACS: Manual Ability Classification System. CFCS: Communication Function Classification System. ASD: Autism spectrum disorder. CBD: Cannabidiol. CLB: clobazam. CBZ: carbamazepine. LAC: lacosamide. LEV: levetiracetam. LTG: lamotrigine. PB: phenobarbital. PER: perampanel. RFM: rufinamide. TPM: topiramate. VNS: Vagus nerve stimulation. VPA: valproic acid. Age in the table reflects the age at the time of the EEG recording.

### Increased Low-Frequency Power in *STXBP1* Syndrome

**Figure 2:**
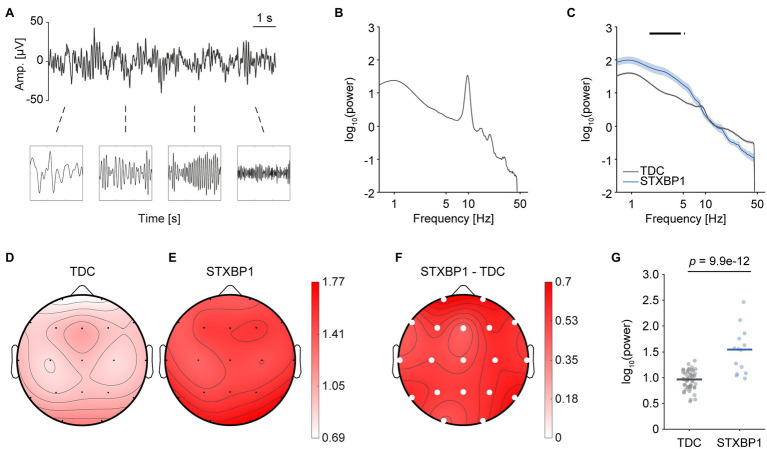
Higher low-frequency power in *STXBP1* syndrome. **(A)** EEG records oscillations with power varying across frequency. **(B)** The power spectrum was computed using the *Welch* method with a Hamming window and a frequency resolution of 0.125 Hz. A typical example of a TDC with a clear 10-Hz alpha peak is shown. **(C)** Whole-brain average power spectrum of TDC and *STXBP1* syndrome show higher power in the range 1.75–4.63 Hz in *STXBP1* syndrome. ANCOVA was performed per frequency bin in the range 1–45 Hz and Bonferroni-corrected across the 353 frequency bins ($p<\frac{.05}{353}$, *black bars*). Shaded areas show standard error of the mean (SEM). **(D–F)** Scalp topography of power averaged across 1.75–4.63 Hz for TDC **(D)**, *STXBP1* syndrome **(E)** and *STXBP1* syndrome minus TDC **(F)**. Low-frequency power is higher in *STXBP1* syndrome at all electrodes. Solid white circles: *p*-values of factor *group* of the ANCOVA with age as a covariate, Bonferroni-corrected by the number of electrodes, $p<\frac{.05}{19}$. **(G)** Whole-brain average power within the range 1.75–4.63 Hz was increased for *STXBP1* syndrome.

First, we evaluated whether heterozygous *STXBP1* variants lead to network-level changes in spectral content of the EEG. To this end, spectral power was compared between *STXBP1* syndrome and TDC (Figures 2A,B). Since there was a similar effect of increased low-frequency power across all electrodes, whole-brain power was computed by taking the mean across electrodes. Whole-brain power was significantly higher for *STXBP1* syndrome in the range 1.75–4.63 Hz (Figure 2C). We then asked whether the elevated power within this frequency range was distributed across the cortex or spatially localized above specific brain areas. The scalp topographies (Figures 2D–F) indicated that increased power in the range 1.75–4.63 Hz was pronounced across the entire cortex [*F*(1,60) = 70.7, *p* = 9.9e-12; Figure 2G]. There was a significant effect of age on power within the range 1.75–4.63 Hz [*F*(1,60) = 13.7, *p* = 4.6e-4]. The developmental profile of low-frequency power was altered in *STXBP1* syndrome: while low-frequency power decreased with age in TDC [linear regression; slope = −0.04, *t*(48) = −8.35, *p* = 6.5e-11], power remained high in children with *STXBP1* syndrome [slope = 0.003, *t*(12) = 0.08, *p* = 0.94; Supplementary Figure 6A]. A traditional frequency-band analysis also revealed increased low-frequency power in *STXBP1* syndrome (Supplementary Figure 3). Taken together, these results show that spectral power of low frequencies was increased in *STXBP1* syndrome and this effect was observed across the cortex.

### Stronger Long-Range Temporal Correlations in *STXBP1* Syndrome

**Figure 3:**
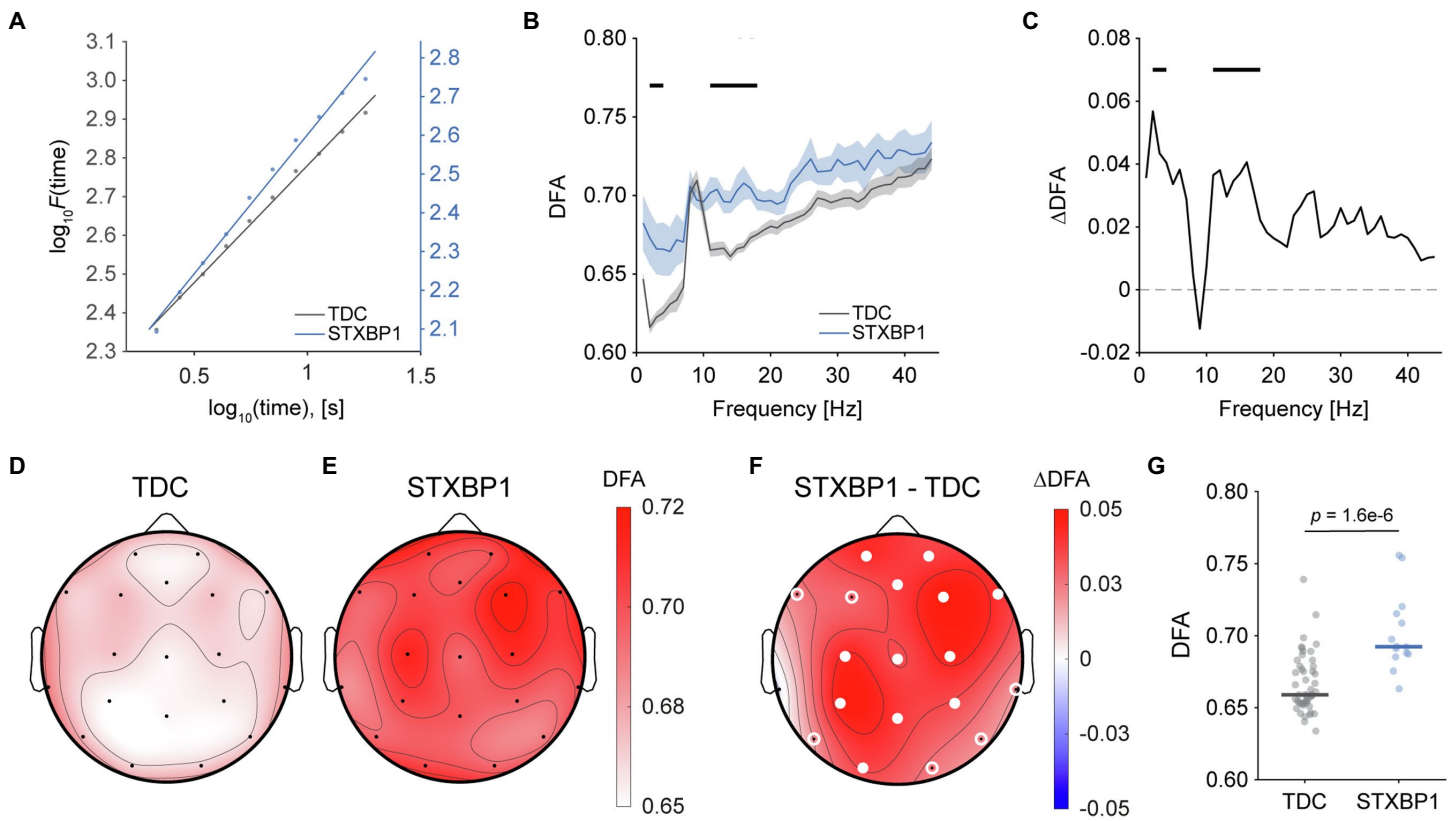
Stronger long-range temporal correlations in *STXBP1* syndrome. **(A)** The DFA exponent is the slope of the log–log linear fits shown, and was used to quantify long-range temporal correlations of brain oscillations. The fit is shown for a typical TDC and *STXBP1* syndrome patient. The y-axis of the *STXBP1* syndrome patient (blue axis) has been offset to highlight the difference in the DFA exponent compared with TDC. **(B)** DFA spectrum in the range 1–45 Hz shows higher DFA exponents for *STXBP1* syndrome in the range 11–18 Hz, indicating stronger LRTC. Black bars: Bonferroni-corrected across frequencies, $p<\frac{.05}{44}$. Confidence intervals show SEM. The spectrum shows the average DFA exponent across subjects and electrodes. **(C)** DFA difference spectrum, computed as *STXBP1* syndrome minus TDC. **(D–F)** Scalp topographies of DFA exponents averaged in the range 11–18 Hz for TDC (D), *STXBP1* syndrome (E), and for *STXBP1* syndrome minus TDC (F). DFA was higher for *STXBP1* syndrome across the cortex. White circles indicate significance based on *p*-values of factor *group* of the ANCOVA with age as a covariate; open white-circles: *p* < 0.05; solid white circles: Bonferroni-corrected,$\;p<\frac{.05}{19}$. **(G)** Whole-brain average DFA in the range 11–18 Hz was higher for *STXBP1* syndrome compared with TDC.

Next, to investigate whether variants in *STXBP1* affect the temporal structure of EEG signals, detrended fluctuation analysis (DFA) was used to quantify long-range temporal correlations (LRTC; Linkenkaer-Hansen et al., 2001; Hardstone et al., 2012; Figure 3A). The whole-brain average DFA exponent was visualized across frequencies in the range 1–45 Hz to yield an LRTC spectrum (Figure 3B). LRTC was significantly stronger in *STXBP1* syndrome for frequency bins in the range 2–3 Hz and 11–18 Hz (Figures 3B,C). LRTC for *STXBP1* syndrome within the range 11–18 Hz was stronger across the cortex [*F*(1,60) = 28.3, *p* = 1.6e-6; Figures 3D–F showing scalp topographies; Figure 3G showing whole-brain average]. There was no significant effect of age [*F*(1,60) = 0.2, *p* = 0.68]. DFA did not significantly change with age in TDC [slope = 0.001, *t*(48) = 1.23, *p* = 0.22] or in *STXBP1* syndrome [slope = −0.002, *t*(12) = −0.95, *p* = 0.36; Supplementary Figure 6B]. Taken together, the temporal structure of the EEG signal in *STXBP1* syndrome is altered compared to controls, showing stronger LRTC across the cortex.

### Decreased *fE/I* in *STXBP1* Syndrome

**Figure 4:**
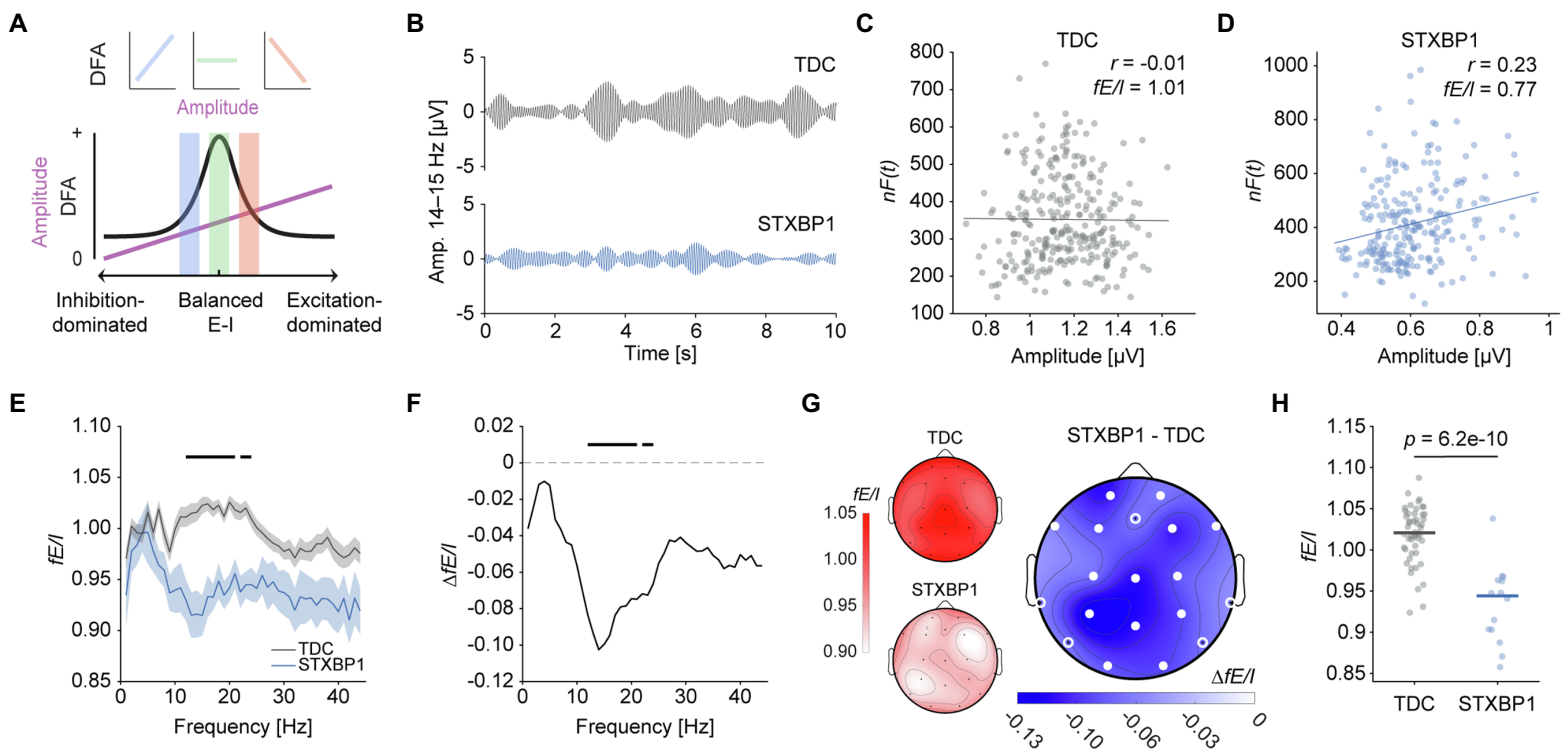
Inhibition-dominated network dynamics in *STXBP1* syndrome. **(A)** Relationship between E/I ratio, DFA, and amplitude in the Critical Oscillations (CROS) model, adapted from Bruining et al. (2020) with permission. An inhibition-dominated regime is characterized by *fE/I* < 1, an excitation-dominated state *fE/I* > 1, and *fE/I* ≈ 1 corresponds to an E/I-balanced network state. Here, *fE/I* was computed in frequency bins of 1 Hz in the range 1–45 Hz, resulting in an *fE/I* spectrum to investigate potential frequency-specificity of E/I-imbalanced network activity. **(B–D)** From a technical perspective, *fE/I* quantifies the covariance between amplitude and a normalized fluctuation function that approximates the DFA exponent of brain oscillations (Bruining et al., 2020). Example signals of a TDC and patient with *STXBP1* syndrome for electrode Pz, FIR-filtered for 14–15 Hz **(B)** and the corresponding Pearson correlation between amplitude and normalized fluctuations **(C,D)**. *fE/I* is computed as 1 minus this correlation. **(E)** *fE/I* spectrum in the range 1–45 Hz averaged across subjects and electrodes shows lower *fE/I* in the range 12–24 Hz in *STXBP1* syndrome. Black bars: Bonferroni-corrected across frequencies, $p<\frac{.05}{44}$. Confidence intervals show SEM. **(F)** *fE/I* difference spectrum of *STXBP1* syndrome minus TDC. **(G)** Scalp topographies of *fE/I* values averaged across 12–24 Hz for TDC (*left top inset*), *STXBP1* syndrome (*left bottom inset*) and *STXBP1* syndrome minus TDC. *fE/I* is lower for *STXBP1* syndrome compared to TDC across the cortex. White circles indicate significance based on *p*-values of factor *group* of the ANCOVA with age as a covariate; open white-circles indicate *p* < 0.05, solid white circles indicate significance after Bonferroni correction for the number of channels, $p<\frac{.05}{19}$. **(H)** In *STXBP1* syndrome, the whole-brain average *fE/I* in the 12–24 Hz range was generally below 1 and lower than TDC, suggesting more inhibition-dominated network activity.

We then tested the hypothesis that *STXBP1* variants lead to network-level changes in E/I balance. First, E/I ratio was assessed using *fE/I*, which quantifies the covariance between amplitude and normalized fluctuations of brain oscillations (Bruining et al., 2020). Previously, we showed that *fE/I* is balanced in healthy individuals and is reduced upon administration of the GABA_A_-receptor agonist zolpidem (Bruining et al., 2020). Hence, we expected *fE/I* to indicate balanced network activity in TDC and E/I disturbances in *STXBP1* syndrome. For E/I-balanced network activity, no correlation between amplitude and fluctuation (*fE/I* ≈ 1) is found, whereas a positive correlation (i.e., *fE/I* < 1) indicates an inhibition-dominated regime and a negative correlation (i.e., *fE/I* > 1) reflects an excitation-dominated state (Figure 4A; Bruining et al., 2020). Indeed, a typical TDC recording (Figure 4B, top) showed no correlation between amplitude and the *fE/I* fluctuation function and thus, an *fE/I* value close to 1 (Figure 4C). In contrast, a representative *STXBP1* syndrome patient (Figure 4B, bottom) showed a positive correlation and therefore an *fE/I* value less than one (Figure 4D). At the group level, *fE/I* was lower in *STXBP1* syndrome compared to TDC across a wide range of frequencies (Figures 4E,F). Similar to spectral power and DFA, the reduction of *fE/I* was widespread across the cortex [*F*(1,60) = 54.1, *p* = 6.2e-10; Figure 4G]. Whole-brain *fE/I* was significantly lower in *STXBP1* syndrome in the range 12–24 Hz (Figure 4H). There was no significant effect of age [*F*(1,60) = 0.4, *p* = 0.54]. *fE/I* in the range 12–24 Hz did not significantly change with age in TDC [slope = −0.002, *t*(48) = −1.26, *p* = 0.39] or in *STXBP1* syndrome [slope = 0.002, *t*(12) = 0.62, *p* = 0.55; Supplementary Figure 6C]. Taken together, these findings suggest that E/I balance is shifted towards stronger inhibition in *STXBP1* syndrome in the 12–24 Hz range.

### Larger Aperiodic Exponents in *STXBP1* Syndrome

**Figure 5:**
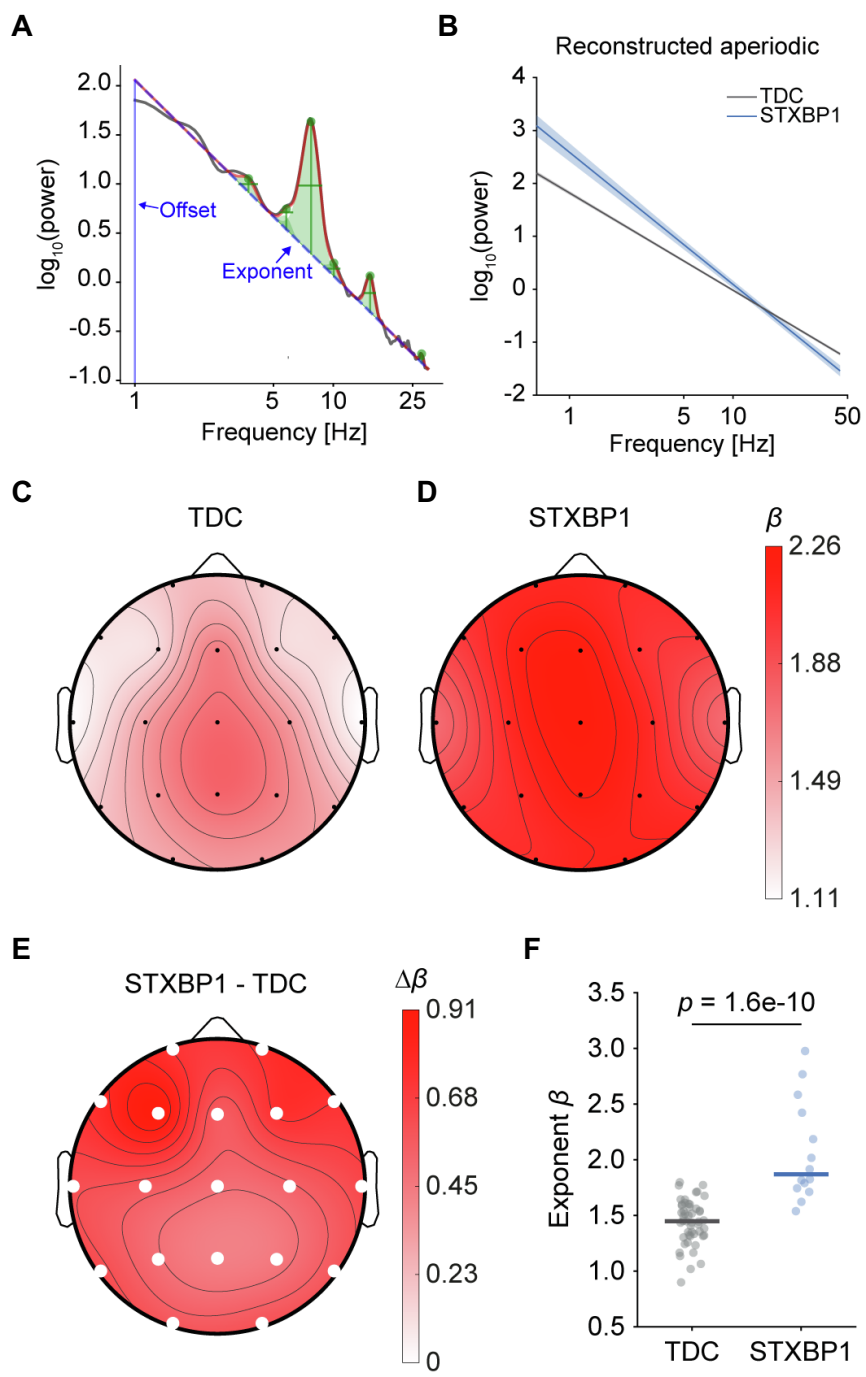
Larger aperiodic exponents in *STXBP1* syndrome. **(A)** The *FOOOF* algorithm was used to estimate the *β* exponent of the 1/f*^β^* aperiodic component of the power spectrum in the range 1–30 Hz (*blue dashed line*). **(B)** Comparison of aperiodic components at channel Pz averaged across participants for TDC and *STXBP1* syndrome. Aperiodic offset and exponent were used to reconstruct an ‘aperiodic-only’ spectrum where oscillatory peaks were removed. Shaded areas show standard error of the mean (SEM). **(C–E)** Scalp topographies of the aperiodic exponents in TDC (C), in *STXBP1* syndrome (D), and *STXBP1* syndrome minus TDC (E). The larger aperiodic exponent in *STXBP1* syndrome compared to TDC was significant at all electrodes (solid white circles: *p*-values from factor *group* of the ANCOVA with age as a covariate, Bonferroni-corrected across electrodes, $p<\frac{.05}{19}$). **(F)** Individual whole-brain average exponents show larger exponents in *STXBP1* syndrome.

As a second measure of E/I balance, the aperiodic exponent was quantified using the *FOOOF* approach (Figure 5A; Donoghue et al., 2020). Recently, the aperiodic exponent has been shown to associate with E/I ratio *in silico*, with larger exponents being associated with lower E/I ratio (Gao et al., 2017). The aperiodic component was reconstructed for each recording and the exponent was extracted. The aperiodic exponent was significantly larger in *STXBP1* syndrome (Figure 5B) across the cortex [*F*(1,60) = 59.4, *p* = 1.6e-10; Figures 5C–E showing scalp topographies, Figure 3F showing whole-brain average]. There was no significant effect of age [*F*(1,60) = 0.2, *p* = 0.69]. Since *FOOOF* removes the spectral peaks before fitting the 1/f-line to the power spectrum, increased aperiodic exponents most likely were not driven by increased low-frequency power. However, it can be argued that large peaks at the start or end of the fitting range may still affect the exponent, even after removing the spectral peaks. Therefore, we investigated whether aperiodic exponents were increased when fitting outside of the range of increased spectral power. When fitted in the range 5–30 Hz, the aperiodic exponent was significantly larger in *STXBP1* syndrome across the cortex [*F*(1,60) = 36.5, *p* = 1.0e-7], without a significant effect of age [*F*(1,60) = 1.1, *p* = 0.31; data not shown]. Aperiodic exponent did not significantly change with age in TDC [slope = −0.01, *t*(48) = −1.82, *p* = 0.08], or in *STXBP1* syndrome [slope = 0.03, *t*(12) = 0.90, *p* = 0.38; Supplementary Figure 6D]. In line with the results for *fE/I*, these findings indicate that E/I balance is shifted towards stronger inhibition in *STXBP1* syndrome.

### Delta Power and Aperiodic Exponents Correlate With Clinical Severity

**Figure 6:**
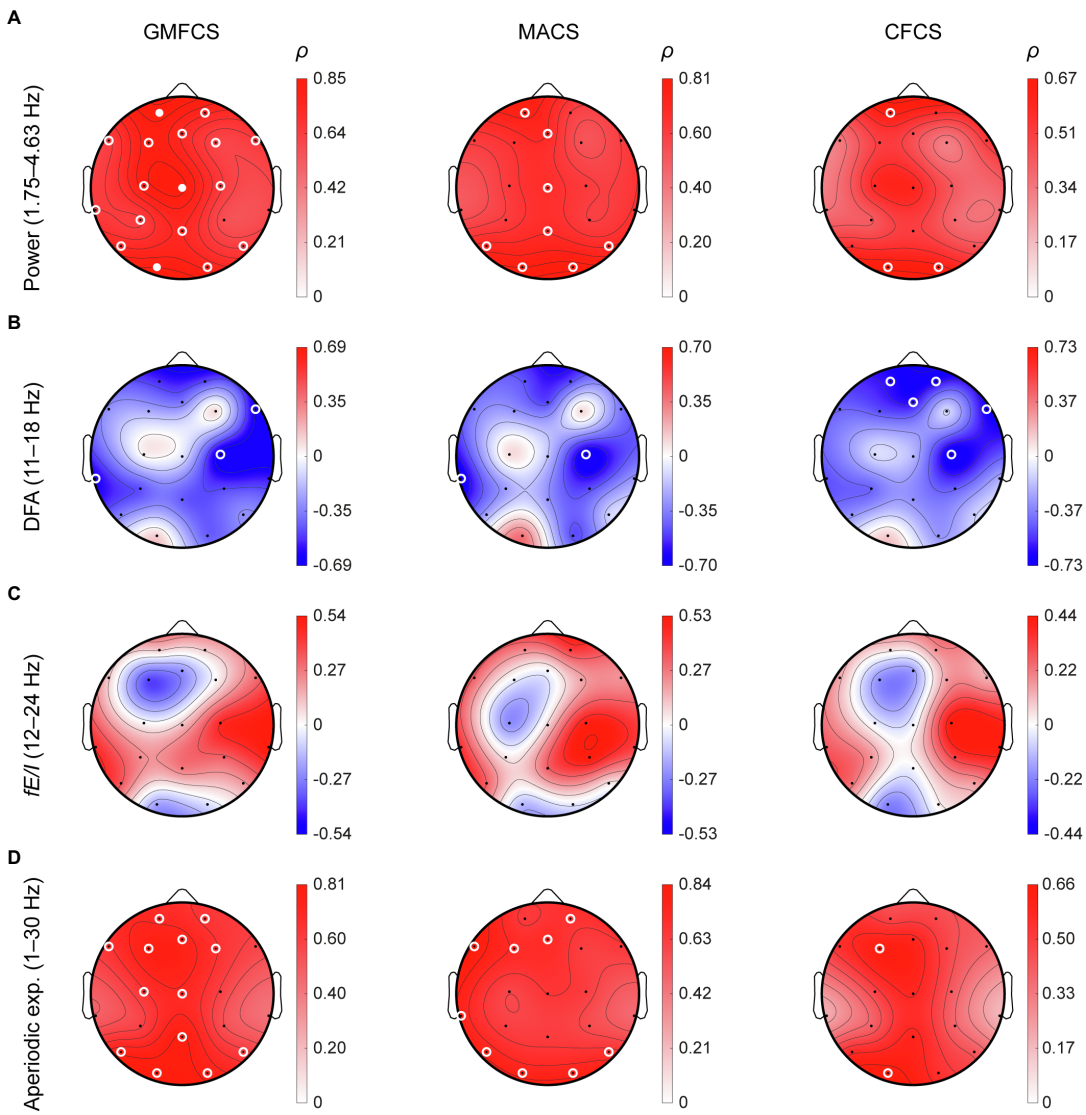
Correlations of EEG parameters with clinical scales. For the *STXBP1* syndrome patients with clinical scores of motor-symptom severity, we computed a partial correlation (*ρ*) between clinical scales and **(A)** power (1.75–4.63 Hz), **(B)** DFA (11–18 Hz), **(C)** *fE/I* (12–24 Hz), and **(D)** aperiodic exponent (1–30 Hz), while controlling for the effect of age. Clinical scales were Gross-Motor Functional Classification System (GMFCS, *n* = 12), Manual Ability Classification System (MACS, *n* = 10) and Communication Functional Classification System (CFCS, *n* = 12). Open white-circles indicate *p* < 0.05, solid white circles indicate significance after Bonferroni correction for the number of channels, $p<\frac{.05}{19}$).

Finally, we investigated whether the observed differences in power, LRTC and *fE/I* in *STXBP1* syndrome were correlated with clinical severity. To test this, EEG parameters were correlated with three clinical scales: GMFCS, MACS, and CFCS, for the *STXBP1* syndrome cases for which these scores were available (*n* = 12; Figure 6). A partial correlation was used to measure the degree of association between each EEG metric and each clinical scale while controlling for the effect of age. Spectral power in the range 1.75–4.63 Hz was positively correlated with clinical severity across the cortex (Figure 6A). DFA in the range 11–18 Hz was negatively correlated with clinical severity at several electrodes (Figure 6B). *fE/I* in the range 12–24 Hz was not significantly associated with the clinical scales (Figure 6C). The aperiodic exponent was positively correlated with GMFCS for several frontal and parietal electrodes (Figure 6D). Taken together, these findings show that higher delta power and larger aperiodic exponents observed in this study are associated with clinical severity in *STXBP1* syndrome.

### Findings Are Unlikely to Be Explained by Differences in Sampling Rate, Recording Setup, or Medication Status at the Time of Recording

Sampling rate should not affect spectral and temporal properties of EEG signals; however, to verify this empirically, we performed our analyses after down-sampling all recordings to a common sampling rate of 200 Hz. The Pearson correlation between recordings with original sampling rate and resampling to 200 Hz was 0.997 for spectral power, 0.998 for DFA, 0.99 for *fE/I*, and 0.9998 for aperiodic exponent. Resampling to a common sampling rate of 200 Hz did not affect our findings (Supplementary Figure 4).

To investigate whether differences in recording setup affected our results, independent samples *t*-test was used to compare EEG measures between the two TDC cohorts and between the three *STXBP1* syndrome cohorts. For power in the range 1.75–4.63 Hz, there was a significant difference between the two TDC cohorts [*t*(48) = 6.7, *p* = 2.3e-8; Supplementary Figure 5A]. Aside from this, no significant differences were observed for spectral power between the three *STXBP1* syndrome cohorts. For DFA (range 11–18 Hz) and *fE/I* (range 12–24 Hz), there were no differences between the two TDC cohorts or between any of the three *STXBP1* syndrome cohorts (Supplementary Figures 5B–D). For the aperiodic exponent, there was no significant difference between the two TDC cohorts, however, there was a significant difference between the *STXBP1* syndrome cohorts from the Netherlands and Denmark [*t*(9) = 2.9, *p* = 0.02], however, not after Bonferroni correction (Supplementary Figure 5D). Taken together, within-group comparisons demonstrate that the four EEG measures are not profoundly affected by recording setup, indicating that heterogeneity in recording sites is unlikely to have driven the group-wise differences observed in this study.

Finally, we assessed whether medication use at the time of recording could explain the differences in EEG measures between TDC and *STXBP1* syndrome. To this end, the *STXBP1* syndrome group was split into patients that were using anti-epileptic medication and patients not using medication (Supplementary Figures 5E–H). For power, DFA, *fE/I* and aperiodic exponent, the no-medication and medication group were both significantly different from TDC. For all four measures assessed here, there was no significant difference between patients without medication and patients with medication after Bonferroni correction (Supplementary Figures 5E–H). These findings indicate that the large differences in EEG measures found between TDC and *STXBP1* syndrome were not explained by medication status at the time of recording.

## Discussion

In this first comprehensive EEG study of an international cohort of patients with *STXBP1* syndrome, we tested the hypothesis that *STXBP1* haploinsufficiency affects resting-state EEG equivalents of network-level E/I balance. We show that *STXBP1* syndrome patients exhibit spatially widespread abnormalities in resting-state EEG measures that indicate inhibition-dominated brain dynamics, attested by (i) increased low-frequency power, (ii) stronger LRTC, (iii) decreased functional *fE/I* ratio, and (iv) increased aperiodic exponents. Importantly, since *STXBP1* syndrome is a developmental disorder and because of heterogeneity in age for both patients and controls, age was included as a covariate in all statistical analyses and the developmental trajectories of these four measures were assessed. Of our main findings, increased low-frequency power and increased aperiodic exponents were associated with clinical severity scores, indicating that the observed EEG findings may have clinical utility.

### Multiple EEG Measures Point to Stronger Network-Level Inhibition in *STXBP1* Syndrome

EEG studies on rare genetic neurodevelopmental disorders as presented here are needed to leverage mechanistic insights on E/I balance dysregulation from preclinical models towards clinical utility. The interpretation of observed effects in EEG studies is often complicated by the fact that the effect of genetic mutations that underlie seizure susceptibility and intellectual disability on functional brain development is as of yet unelucidated. In the case of *STXBP1* syndrome, we show that this genetic disorder leads to global slowing and inhibition-dominated activity as reflected in our quantitative analysis of resting-state EEG measurements. Notably, the observed effect directions are highly homogeneous, with all patients displaying highly similar alterations compared to controls, despite the substantial genetic heterogeneity that is characteristic for *STXBP1* syndrome, observed in the cohort. This indicates that the EEG alterations are general to *STXBP1* syndrome patients.

Firstly, delta power (1.75–4.63Hz) is increased in *STXBP1* syndrome patients: in typically developing children, delta-range power decreases with age, in line with literature (Clarke et al., 2001), whereas it remains high in *STXBP1* syndrome patients. The observed increased power is similar to the increased power of delta oscillations during wakefulness that has been observed in a variety of neuropathologies (Knyazev, 2012; Wang et al., 2013) and is often associated with epilepsy and epileptiform brain activity (Gambardella et al., 1995; Geyer et al., 1999; Clemens et al., 2000; Huppertz et al., 2001; Baayen et al., 2003; Schönherr et al., 2017; Nissen et al., 2018). In healthy individuals, delta oscillations predominantly occur during slow-wave sleep (Silber et al., 2007), during which neurons are globally inhibited by GABA (Hobson and Pace-Schott, 2002). In addition, delta power is increased during anesthesia (Chennu et al., 2014) and in comatose patients (Kaplan, 2004). Taken together, the observed increase in delta power in *STXBP1* syndrome patients suggests a slowing and shift in brain activity towards a more inhibition-dominated regime.

Secondly, stronger LRTC in the range 11–18 Hz of the *STXBP1* syndrome patients is in line with increased LRTC observed in intracranially recorded oscillations around seizure loci in patients with epilepsy (Parish et al., 2004; Stead et al., 2005; Monto et al., 2007). Increased LRTCs may result from the presence of intermittent inter-ictal spiking activity, burst-suppression patterns, or hypsarrhythmia (Parish et al., 2004). Such EEG abnormalities are frequently observed in *STXBP1* syndrome patients (Stamberger et al., 2016), e.g., burst-suppression patterns. Burst-suppression patterns are characterized by periods of high-voltage alternated by low-voltage electrical activity (Steriade et al., 1994) and also observed during conditions of profoundly inhibition-dominated brain state, such as anesthesia and coma (Brown et al., 2010).

Thirdly, our recently developed *fE/I* measure is lower in patients with *STXBP1* syndrome compared to TDC. Previously, it was shown that *fE/I* is balanced (i.e., *fE/I* ≈ 1) in healthy adults, which is also observed in the present study for typically developing children, and is reduced during administration of zolpidem, a GABA_A_-receptor agonist (Bruining et al., 2020). The decrease of *fE/I* in *STXBP1* syndrome was observed in the frequency range of 12–24 Hz, which is a frequency range that has previously been associated with GABAergic signaling (Feshchenko et al., 1997; Jensen et al., 2005; Frohlich et al., 2016, 2019), and which approximates the frequency range in which LRTC alterations are observed (beta range). Interestingly, we have previously shown that decreased *fE/I* in children with autism was most evident in the subset of patients with comorbid EEG abnormalities (Bruining et al., 2020). Hence, it is possible that either, EEG abnormalities in *STXBP1* syndrome also drive the reduction in *fE/I*, or that synaptic changes drive both the occurrence of EEG abnormalities and a reduction in *fE/I*.

Finally, increased aperiodic exponents of spectral power are observed in *STXBP1* syndrome. Power spectra of brain oscillations contain an aperiodic component characterized by a $1/f^{\beta }$ relationship between power and frequency (Freeman and Zhai, 2009; He, 2014), on top of which oscillatory rhythms give rise to peaks (Donoghue et al., 2020). Reducing synaptic E/I ratio *in silico* leads to a larger aperiodic exponent of spectral power (Gao et al., 2017). In empirical data, an increased aperiodic exponent has been observed in brain states characterized by increased neuronal inhibition including sleep (He et al., 2010; Miskovic et al., 2019; Lendner et al., 2020) and anesthesia (Gao et al., 2017; Colombo et al., 2019). Therefore, the increased aperiodic exponent provides further evidence for the notion of a shift towards inhibition-dominated brain dynamics in *STXBP1* syndrome patients.

Taken together, multiple lines of evidence indicate that in patients harboring an *STXBP1* variant, brain activity is shifted toward an inhibition-dominated state compared to TDC. Changes in EEG parameters occur in similar frequency ranges that overlap with the beta frequency range, that has been associated with GABAergic signaling, and thus may reflect changes in E/I ratio at the synapse level.

### E/I Disturbances in *STXBP1* Syndrome May Reflect a Compensatory Mechanism in Response to Local Circuitry Imbalances

Increased inhibition in a disorder strongly associated with epileptic seizures may seem counterintuitive, considering that excess of excitation is commonly believed to underlie ictogenesis (Treiman, 2001; Galanopoulou, 2010). However, the assumption of hyperexcitability underlying ictogenesis is based on seizure activity itself and on preclinical findings in cellular models. Less is known about the excitation-inhibition balance of mass-neuronal activity during inter-ictal periods, which are analyzed in resting-state EEG. Findings in rodent models of *STXBP1* haploinsufficiency have provided evidence for E/I imbalance in local brain circuitries. In rodent neurons, inhibitory neurotransmission was found to be reduced at the single-neuron level *in vitro* (Toonen et al., *2006**)* and within cortico-cortical circuits of *STXBP1* haploinsufficient mice (Chen et al., 2020), whereas excitatory cortico-striatal projections were found to have reduced synaptic strength, leading to E/I imbalance in cortico-striatal and cortico-thalamic circuitries (Miyamoto et al., 2019).

Due to the fundamental nature of E/I balance for neural network organization and information processing and its tight regulation in the human brain (Shu et al., 2003; Turrigiano and Nelson, 2004; Haider et al., 2006; Kinouchi and Copelli, 2006; Dehghani et al., 2016), local circuitry imbalances are likely to be compensated in an attempt to restore network homeostasis. E/I ratio may be regulated at the synaptic level or through altering network connectivity (Van Vreeswijk and Sompolinsky, 1996; Turrigiano and Nelson, 2004; Carcea and Froemke, 2013; Nelson and Valakh, 2015). Here, in contrast to measurements in specific local circuits in mouse models, the EEG signal reflects the integration of local circuitry imbalances caused by synaptic deficits, possibly further offset by compensatory mechanisms. In the case of *STXBP1* syndrome, we show that this integration culminates in a brain-wide inhibition-dominated state, which may be associated with clinical manifestations relevant to *STXBP1* syndrome. Indeed, there is evidence associating an excess of global inhibition in neural circuits to several developmental intellectual disability syndromes (Fernandez and Garner, 2007; Bexkens et al., 2014). Most of these syndromes have varying degrees of EEG abnormalities and it may be conceivable that inhibition there also is a secondary compensatory phenomenon. Intellectual disability is the most cardinal feature observed in all patients with *STXBP1* syndrome. In line with this reasoning, the shift towards inhibition is observed in both patients with and without epilepsy. Future studies should delineate the exact mechanistic relationship between the different levels of neural organization and may include additional disease groups to discern whether the observed effects are specific to *STXBP1* syndrome or a more general feature of syndromes characterized by similar symptoms.

### Methodological Considerations to Studying Rare Neurodevelopmental Disorders

Studying rare neurodevelopmental disorders poses several challenges: obtaining recordings from different sites with varying equipment; relatively small achievable sample sizes; and EEG recordings in children and adults with combined behavioral and intellectual disabilities. It is nearly impossible for patients to sit still and keep their eyes closed, so EEG was recorded with eyes-open, resulting in frequent movement and eye blink artifacts. To limit artifact biases in statistical outcomes, preprocessing of the recordings was first performed in an unbiased, quantitative manner followed by thorough visual inspection by two EEG experts. On average, 11% of the channels were detected as noisy and interpolated, 9.5% of all 1-s epochs were removed, and two independent components were rejected, which are acceptable amounts of data loss for reliable quantification of EEG parameters that were reported here. Spectral power can reliably be estimated from recordings with a signal length > 20 s (Gasser et al., 1985; Salinsky et al., 1991), although reliability increases up to a signal length of 40 s (Gudmundsson et al., 2007). Here, the minimum signal length to include a recording was 100 s, a threshold that we have found to give a reliable estimation of temporal measures DFA and *fE/I* (unpublished observations).

This study reports on analyses of recordings aggregated from five different recording sites, with varying recording setups, sampling rates, and numbers of electrodes. To avoid that these differences would affect the outcome of the EEG analyses, we selected the 19 channels of the 10–20 international electrode placement from each recording site, i.e., electrodes analyzed were placed at the same scalp locations across EEG recording setups. Theoretically, one would not expect sampling-rate differences to affect spectral power, DFA or *fE/I*. To confirm this, we down-sampled all recordings to the lowest common sampling rate of 200 Hz and compared analysis outcomes, which demonstrated that none of our findings were affected by sampling-rate differences (see Supplementary Figure 4). Notably, the effect direction for increased power (Figure 2G), DFA (Figure 3G), *fE/I* (Figure 4H), and aperiodic exponent (Figure 5F) was the same for all patients (except for the *fE/I* value of one patient; see Figure 4H). This indicates a homogeneous direction of effect of genetic variants on EEG in *STXBP1* syndrome, regardless of heterogeneity across recording setups. Furthermore, DFA is known to be robust across recording sites and setups, because the temporal structure is independent of any potential offsets in amplifier calibration. DFA in the alpha range (8–13 Hz) of participants during eyes-closed rest has consistently been reported to fall in the range 0.65–0.75 (Linkenkaer-Hansen et al., 2001, 2007; Berthouze et al., 2010; Smit et al., 2011; Bruining et al., 2020). Lower DFA values (~0.65) have been reported for young children aged 7–14 years (Bruining et al., 2020), in line with expected DFA values (0.65–0.68 for age range 5–16 years; Smit et al., 2011), which closely matches the DFA values observed in the present study for controls (i.e., 0.66 ± 0.02). Notably, DFA values of 29 controls from Bruining et al. (2020) and 21 controls from Schiavone et al. (2014), that together made up the control sample here, were not significantly different from each other (Supplementary Figure 5B).

Moreover, *fE/I* is relatively insensitive to recording equipment. Previously, *fE/I* within the alpha range (8–13 Hz) was found to be 0.99 ± 0.01 for healthy adults, indicating balanced excitation-inhibition (age range 19–56 years, 176 participants; Bruining et al., 2020), and similarly, *fE/I* within the alpha range for typically developing children was found to be 1.01 ± 0.02 (age range 7–14 years, 29 participants; Bruining et al., 2020). In the present study, *fE/I* of the 21 controls not included previously in the study by Bruining et al. (2020) was again highly comparable within the alpha range (1.03 ± 0.03), as well as within the 12–24 Hz range analyzed here (1.03 ± 0.03), and *fE/I* between those two TDC cohorts was not significantly different (Supplementary Figure 5C). Furthermore, *fE/I* of the TDC cohorts together or separately was not significantly different from a large cohort of 176 recordings of healthy adult individuals reported in Bruining et al. (2020). These findings indicate that *fE/I* is balanced close to 1 in healthy individuals and does not substantially vary across recording site or setup. Therefore, we show that *fE/I* can be analyzed or compared across recordings from different setups.

In contrast, 1/f-like scaling properties of power spectra are sensitive to recording equipment and laboratory and, thus, should be interpreted with caution. However, it should be noted that a larger aperiodic exponent is in line with the reduced *fE/I*, since the effect in both measures indicates a shift towards a more inhibition-dominated brain activity regime.

Furthermore, seven out of 10 patients with epilepsy were using anti-epileptic medications at the time of recording, whereas patients without epilepsy did not. We showed that there were no differences in EEG measures between patients with medication and those without medication, after Bonferroni correction (Supplementary Figure 5). At uncorrected alpha (i.e., *α* = 0.05), *fE/I* was counterintuitively higher for patients using anti-epileptic medication (indicating a less inhibition-dominated brain state), whereas aperiodic exponent was higher, indicating increased inhibition, more in line with the canonically accepted effect of anti-epileptic medications. Notably, these effects were not significant after Bonferroni correction and larger sample sizes are needed to assess the effects of anti-epileptic medication more in-depth. Nevertheless, the absence of significant differences based on medication status lead us to conclude that the observed differences in EEG measures between TDC and *STXBP1* syndrome do not have a pharmacological origin.

As a technical innovation, we investigated for the first time the usefulness of applying DFA and *fE/I* to 1-Hz narrow frequency bins instead of the classical frequency bands (i.e., delta, theta, alpha, and beta bands) to investigate how spectral and temporal dynamics of brain oscillations are affected in disease altered across a wide range of frequencies. The results indicate that this approach – having a high-frequency resolution and being hypothesis-free for the frequency content of aberrant oscillatory activity – is promising for gaining new insights into pathophysiology and should be tested in other clinical studies.

### Conclusion and Future Directions

This first study in a cohort of patients harboring *STXBP1* variants shows that quantitative EEG analysis can form a bridge between preclinical studies on molecular and cellular pathogenic mechanisms on one hand, and clinical parameters on the other, by providing quantitative pathophysiological parameters of whole-brain activity, which are crucial steps towards the development and application of mechanism-based treatments.

## Data Availability Statement

The original contributions presented in the study are included in the article/Supplementary Material; further inquiries can be directed to the corresponding author.

## Ethics Statement

The studies involving human participants were reviewed and approved by the Medical Ethical Committee Amsterdam UMC, location VUmc. Written informed consent to participate in this study was provided by the participants’ legal guardian or next of kin.

## Author Contributions

SH and HL designed the study and drafted the manuscript. SH recorded the Amsterdam sample, wrote the analysis scripts, and performed the data analyses. HL and AB organized the STXBP1-clinic days in the Netherlands. GB, FZ, and PS provided the data for the Italy sample. EG, CR, and RM provided the data for the Denmark sample. MM-I, MH, and ME performed the clinical assessments of patients in the Netherlands. TZ provided the control recordings measured at UvA. HM supervised the project. MV initiated and designed the study and supervised the project. KL-H and HB designed the study and supervised the project. All authors contributed to the article and approved the submitted version.

## Funding

EG, MV, and RM received a grant from the Lundbeck Foundation on “Neurodevelopmental disorders: from synaptic mutation to disease” (grant no. R277-2018-802). MV was supported by a European Research Council Advanced Grant (322966), COSYN (Comorbidity and Synapse Biology in Clinical Overlapping Psychiatric Disorders; Horizon 2020 Program of the European Union under RIA grant agreement 667301 to MV), the NWO Gravitation program grant BRAINSCAPES (NWO 024.004.012) and an MDBR 2019 Pilot Grant from the Orphan Disease Center (MDBR-20-136-STXBP1). HB, KL-H, and MV were supported by the Netherlands Organization for Scientific Research (NWO) Dutch National Research Agenda, NWA-ORC Call (NWA.1160.18.200).

## Conflict of Interest

KL-H is a shareholder of NBT Analytics BV, which provides EEG-analysis services for clinical trials. HB and KL-H are shareholders of Aspect Neuroprofiles BV, which develops physiology-informed prognostic measures for neurodevelopmental disorders. KL-H has filed the patent claim (PCT/NL2019/050167) “Method of determining brain activity”; with priority date 16 March 2018.

The remaining authors declare that the research was conducted in the absence of any commercial or financial relationships that could be construed as a potential conflict of interest.

## Publisher’s Note

All claims expressed in this article are solely those of the authors and do not necessarily represent those of their affiliated organizations, or those of the publisher, the editors and the reviewers. Any product that may be evaluated in this article, or claim that may be made by its manufacturer, is not guaranteed or endorsed by the publisher.

## Supplementary Material

Supplementary Figure 1 Preprocessing pipeline. Recordings were imported into EEGLAB format and bandpass-filtered between 1–45 Hz using a FIR-filter with a Hamming window. Bad channels were defined as electrodes with a power spectrum, joint probability or kurtosis that deviated more than 3 standard deviations from all other channels. Bad channels were interpolated using a spherical spline. To aggregate recordings from different recording sites, 19 channels of the 10–20 international system were selected. Recordings were then segmented into 1-s artificial data blocks. Epochs that exceeded the range [−150, 150] μV were rejected. Independent component analysis (ICA) in combination with *ICLabel* was used to remove components with eye artifacts. All recordings were then scrolled through in windows of 10 s by two EEG experts to remove remaining noise not picked up by the automatic procedure. Epochs that were judged as ‘good’ by both raters were kept for further analysis. Recordings were excluded if the clean signal length was below 100 s or for which more than half of the signal in the temporal domain was removed.

Supplementary Figure 2 Main findings are not driven by preprocessing procedure or signal length. **(A)** Percentage of channels that was interpolated out of the final 19 channels selected for analysis. The topographical inset shows the percentage of subjects for which a specific channel was detected as bad, interpolated between electrodes, showing that noise was mainly positioned at the rim of the head. **(B)** Percentage of bad epochs removed after automatic and manual cleaning. **(C)** Number of eye components rejected by *ICLabel*. **(D)** Final signal length in minutes. **(E)** Typical scalp topographies of horizontal (HEOG, left) and vertical (VEOG, right) eye movements detected by *ICLabel*. All rejected components were examined to confirm that only components with eye artifacts were rejected. **(F)** Heatmap of *F*-values of factor group of an ANCOVA with the value of each whole-brain averaged EEG measure (rows) as the dependent factor, group as the independent factor and processing or signal statistics as continuous covariate (columns). Factor group (i.e., TDC vs. *STXBP1* syndrome) was significant. **(G)** Heatmap of *F*-values of the covariate included in the ANCOVA. In (F) and (G), significance was defined as p<.05, and Bonferroni-corrected for the number of combinations between EEG measures and processing statistics (i.e., $p<\frac{.05}{16}$). * p<.05, ** p<.01, *** p<.001, **** p<.0001, ***** p<.00001.

Supplementary Figure 3 Shift in spectral content indicates slowing of activity in *STXBP1* syndrome. **(A)** EEG measures oscillations with power varying across frequency. Signals were decomposed into four canonical frequency bands using the Fourier transform. **(B)** Power spectral density (PSD) was computed for all recordings and electrodes using the *Welch* method with a Hamming window and a frequency resolution of 0.125 Hz. Frequency bands were defined as delta (1–4 Hz), theta (4–8 Hz), alpha (8–13 Hz) and beta (13–30 Hz). A typical example of a TDC with a clear 10 Hz alpha peak is shown. **(C)** Mean PSD of all TDC (*n* = 50) and *STXBP1* syndrome patients (*n* = 14) show increased power at low frequencies in *STXBP1* syndrome. Shaded areas show standard error of the mean (SEM). **(D)** Topographies of absolute power, relative power, and central frequency for all bands. **(E)** Whole-brain average of power and central frequency show an increase of power within low frequency bands and decreased power within high frequency bands. White circles indicate significance based on *p*-values of a Wilcoxon rank-sum test; open white-circles indicate p<.05, solid white circles indicate significance after Bonferroni correction for the number of channels, $p<\frac{.05}{19}$.

Supplementary Figure 4 Down-sampling to a common sampling rate of 200 Hz did not affect our results. Panels show the same findings as in Supplementary Figures 2–5, after down-sampling the preprocessed recordings to 200 Hz, which was the lowest common sampling rate in the aggregated dataset. **(A)** Spectral power. **(B)** Aperiodic exponent. **(C)** DFA. **(D)** *fE/I*.

Supplementary Figure 5 Comparisons of sub-cohorts indicate homogeneity of EEG findings across sub-cohorts of the same condition and indicate no difference between patients with or without anti-epileptic medication. Independent samples t-test was used to compare whole-brain averaged EEG measures across sub-cohorts. Significance was defined as p<.05, Bonferroni-corrected for the number of comparisons within each panel (i.e., $p<\frac{.05}{4}$). **(A)** Spectral power in the range 1.75–4.63 Hz was significantly different between the two TDC cohorts, but not between any of the three *STXBP1* syndrome cohorts. **(B–D)** There were no significant differences between the two TDC cohorts or between any of the three *STXBP1* syndrome cohorts after Bonferroni correction for DFA in the range 11–18 Hz (B), *fE/I* in the range 12–24 Hz **(C)** or aperiodic exponent fitted in the range 1–30 Hz **(D)**. **(E–H)** Since anti-epileptic medication is thought to reduce excitation, we assessed whether EEG measures from *STXBP1* syndrome patients with or without medication were significantly different from each other. EEG measures were significantly different from TDC for *STXBP1* syndrome patients with or without medication, but not significantly different between the two groups split for medication status. No med: No medication; Med: Medication at the time of recording.

Supplementary Figure 6 Developmental trajectories of EEG measures show altered development of low-frequency power in *STXBP1* syndrome. Linear regression was used to fit a model with the whole-brain average of each EEG metric as dependent variable and age as a predictor. Developmental trajectories for TDC (grey) and *STXBP1* syndrome (blue) for **(A)** Low-frequency power, **(B)** DFA, **(C)** *fE/I*, and **(D)** Aperiodic exponent.
