# Supplementary figures and images for: STXBP1 Syndrome Is Characterized by Inhibition-Dominated Dynamics of Resting-State EEG
Source: Front Physiol. 2021 Dec 23;12:775172. doi: 10.3389/fphys.2021.775172 (PMC8733612; doi:10.3389/fphys.2021.775172)

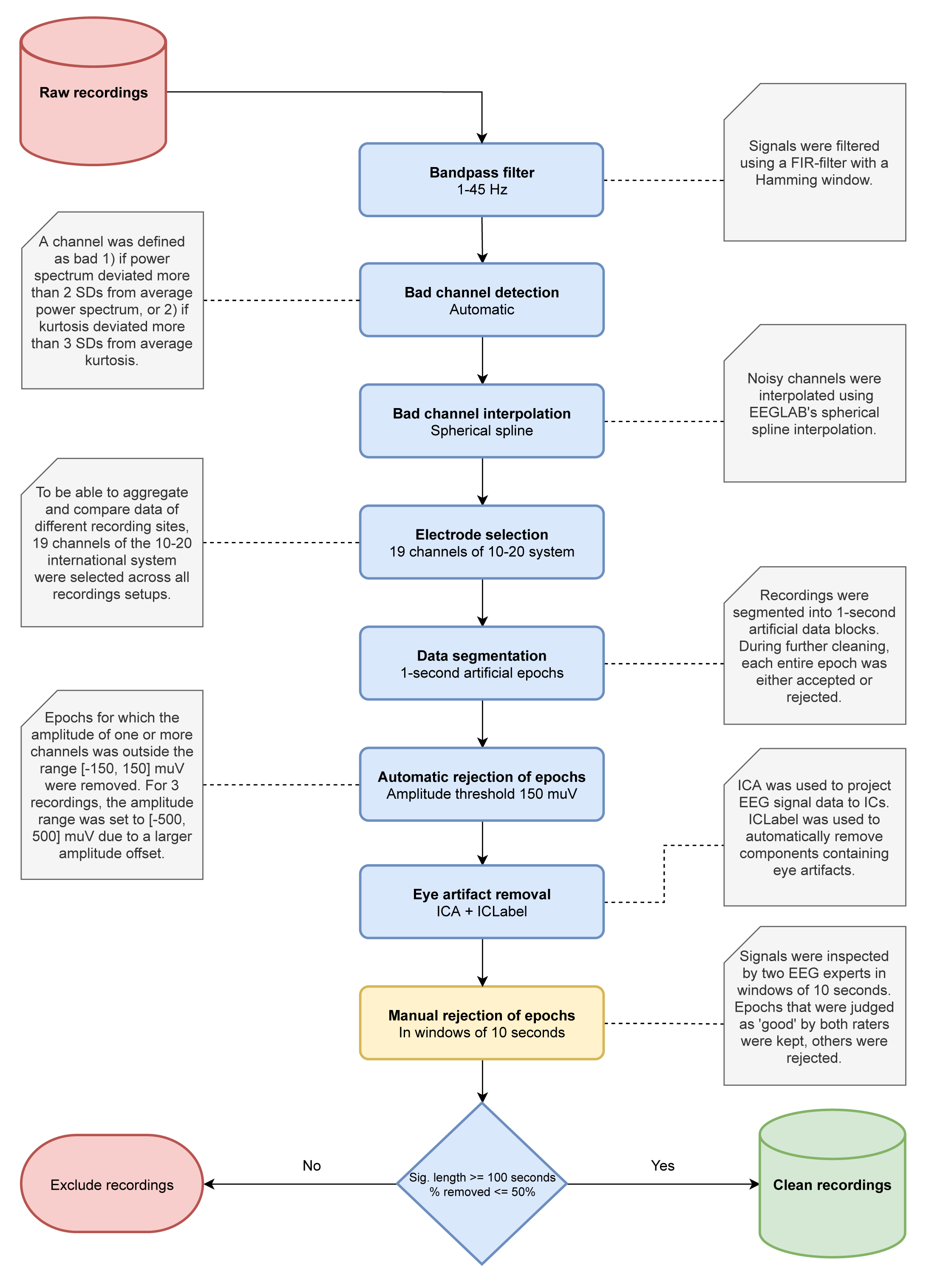

Supplement: Supplementary Figure 1 — Preprocessing pipeline. Recordings were imported into EEGLAB format and bandpass-filtered between 1–45 Hz using a FIR-filter with a Hamming window. Bad channels were defined as electrodes with a power spectrum, joint probability or kurtosis that deviated more than 3 standard deviations from all other channels. Bad channels were interpolated using a spherical spline. To aggregate recordings from different recording sites, 19 channels of the 10–20 international system were selected. Recordings were then segmented into 1-s artificial data blocks. Epochs that exceeded the range [−150, 150] μV were rejected. Independent component analysis (ICA) in combination with ICLabel was used to remove components with eye artifacts. All recordings were then scrolled through in windows of 10 s by two EEG experts to remove remaining noise not picked up by the automatic procedure. Epochs that were judged as ‘good’ by both raters were kept for further analysis. Recordings were excluded if the clean signal length was below 100 s or for which more than half of the signal in the temporal domain was removed. [file Image_1.TIF]

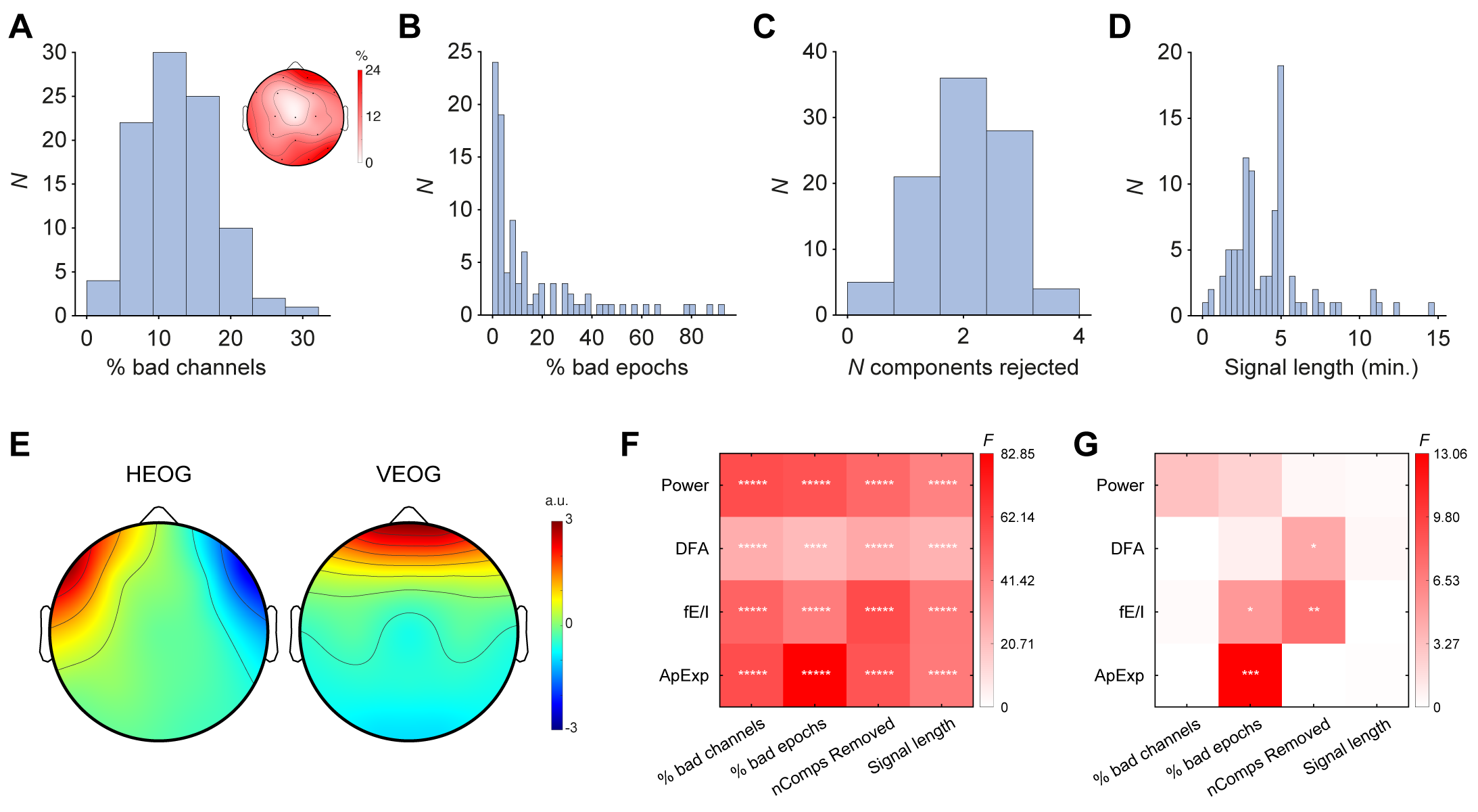

Supplement: Supplementary Figure 2 — Main findings are not driven by preprocessing procedure or signal length. (A) Percentage of channels that was interpolated out of the final 19 channels selected for analysis. The topographical inset shows the percentage of subjects for which a specific channel was detected as bad, interpolated between electrodes, showing that noise was mainly positioned at the rim of the head. (B) Percentage of bad epochs removed after automatic and manual cleaning. (C) Number of eye components rejected by ICLabel. (D) Final signal length in minutes. (E) Typical scalp topographies of horizontal (HEOG, left) and vertical (VEOG, right) eye movements detected by ICLabel. All rejected components were examined to confirm that only components with eye artifacts were rejected. (F) Heatmap of F-values of factor group of an ANCOVA with the value of each whole-brain averaged EEG measure (rows) as the dependent factor, group as the independent factor and processing or signal statistics as continuous covariate (columns). Factor group (i.e., TDC vs. STXBP1 syndrome) was significant. (G) Heatmap of F-values of the covariate included in the ANCOVA. In (F) and (G), significance was defined as p<.05, and Bonferroni-corrected for the number of combinations between EEG measures and processing statistics (i.e., p<.0516). * p<.05, ** p<.01, *** p<.001, **** p<.0001, ***** p<.00001. [file Image_2.TIF]

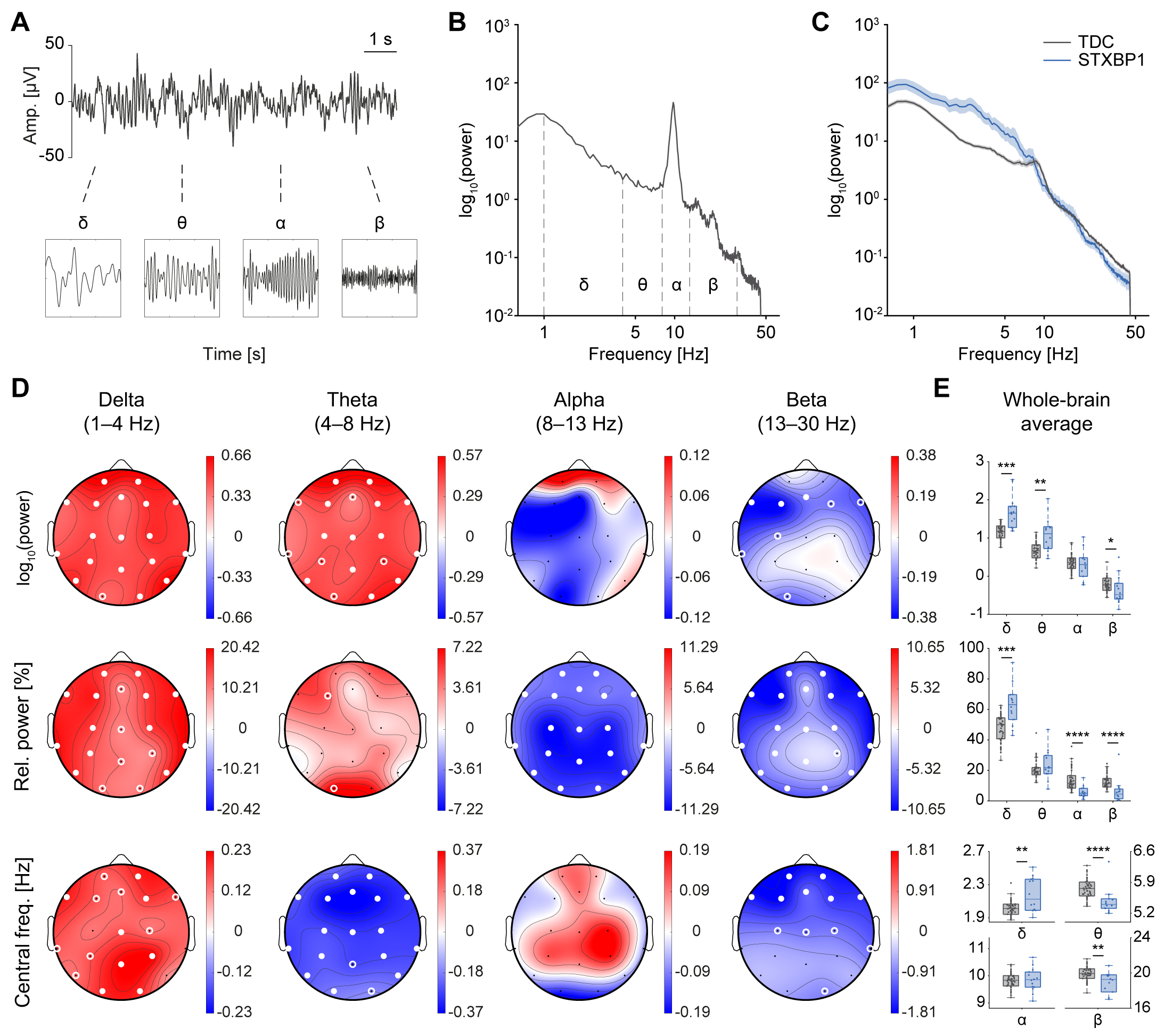

Supplement: Supplementary Figure 3 — Shift in spectral content indicates slowing of activity in STXBP1 syndrome. (A) EEG measures oscillations with power varying across frequency. Signals were decomposed into four canonical frequency bands using the Fourier transform. (B) Power spectral density (PSD) was computed for all recordings and electrodes using the Welch method with a Hamming window and a frequency resolution of 0.125 Hz. Frequency bands were defined as delta (1–4 Hz), theta (4–8 Hz), alpha (8–13 Hz) and beta (13–30 Hz). A typical example of a TDC with a clear 10 Hz alpha peak is shown. (C) Mean PSD of all TDC (n = 50) and STXBP1 syndrome patients (n = 14) show increased power at low frequencies in STXBP1 syndrome. Shaded areas show standard error of the mean (SEM). (D) Topographies of absolute power, relative power, and central frequency for all bands. (E) Whole-brain average of power and central frequency show an increase of power within low frequency bands and decreased power within high frequency bands. White circles indicate significance based on p-values of a Wilcoxon rank-sum test; open white-circles indicate p<.05, solid white circles indicate significance after Bonferroni correction for the number of channels, p<.0519. [file Image_3.TIF]

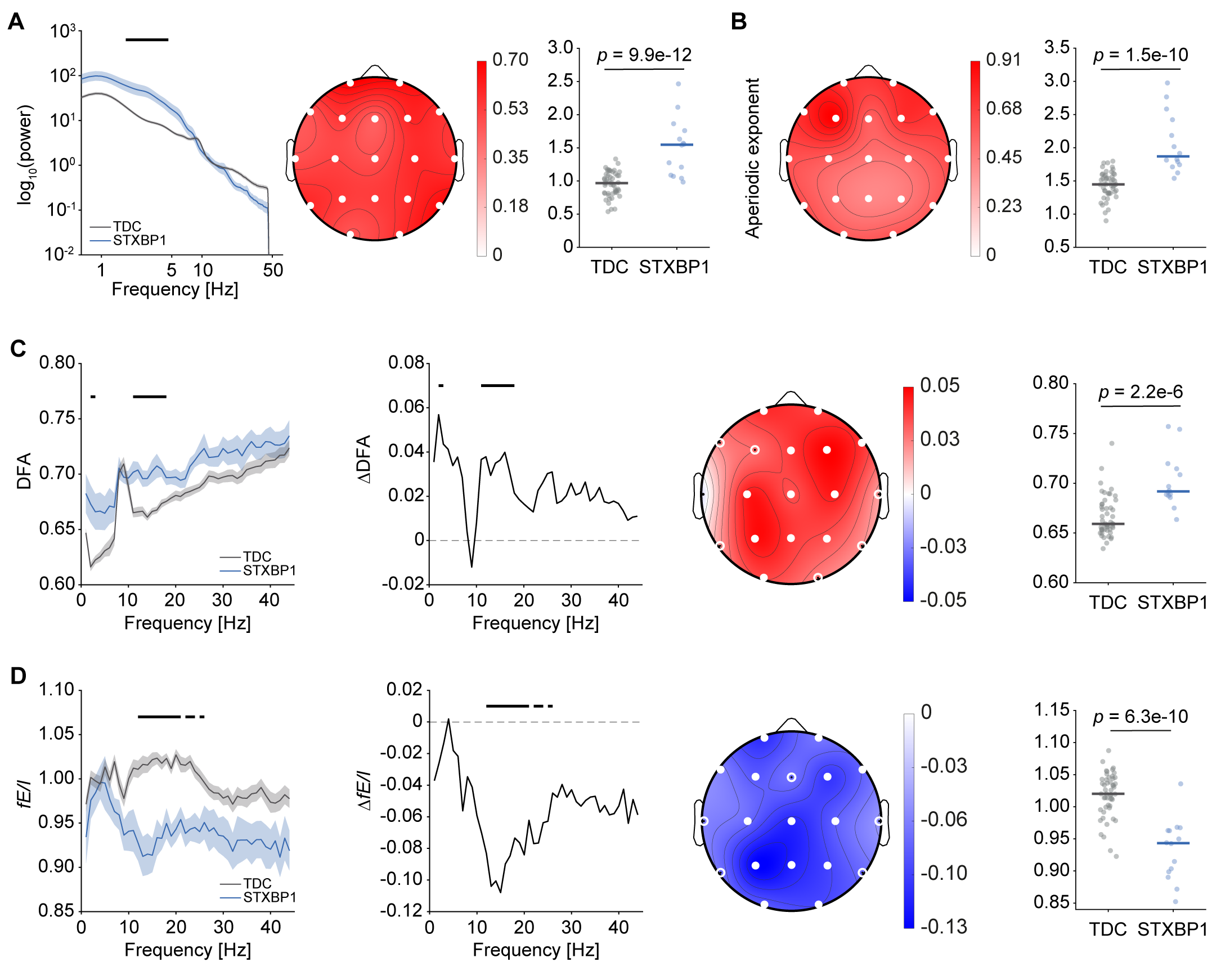

Supplement: Supplementary Figure 4 — Down-sampling to a common sampling rate of 200 Hz did not affect our results. Panels show the same findings as in Supplementary Figures 2–5, after down-sampling the preprocessed recordings to 200 Hz, which was the lowest common sampling rate in the aggregated dataset. (A) Spectral power. (B) Aperiodic exponent. (C) DFA. (D) fE/I. [file Image_4.TIF]

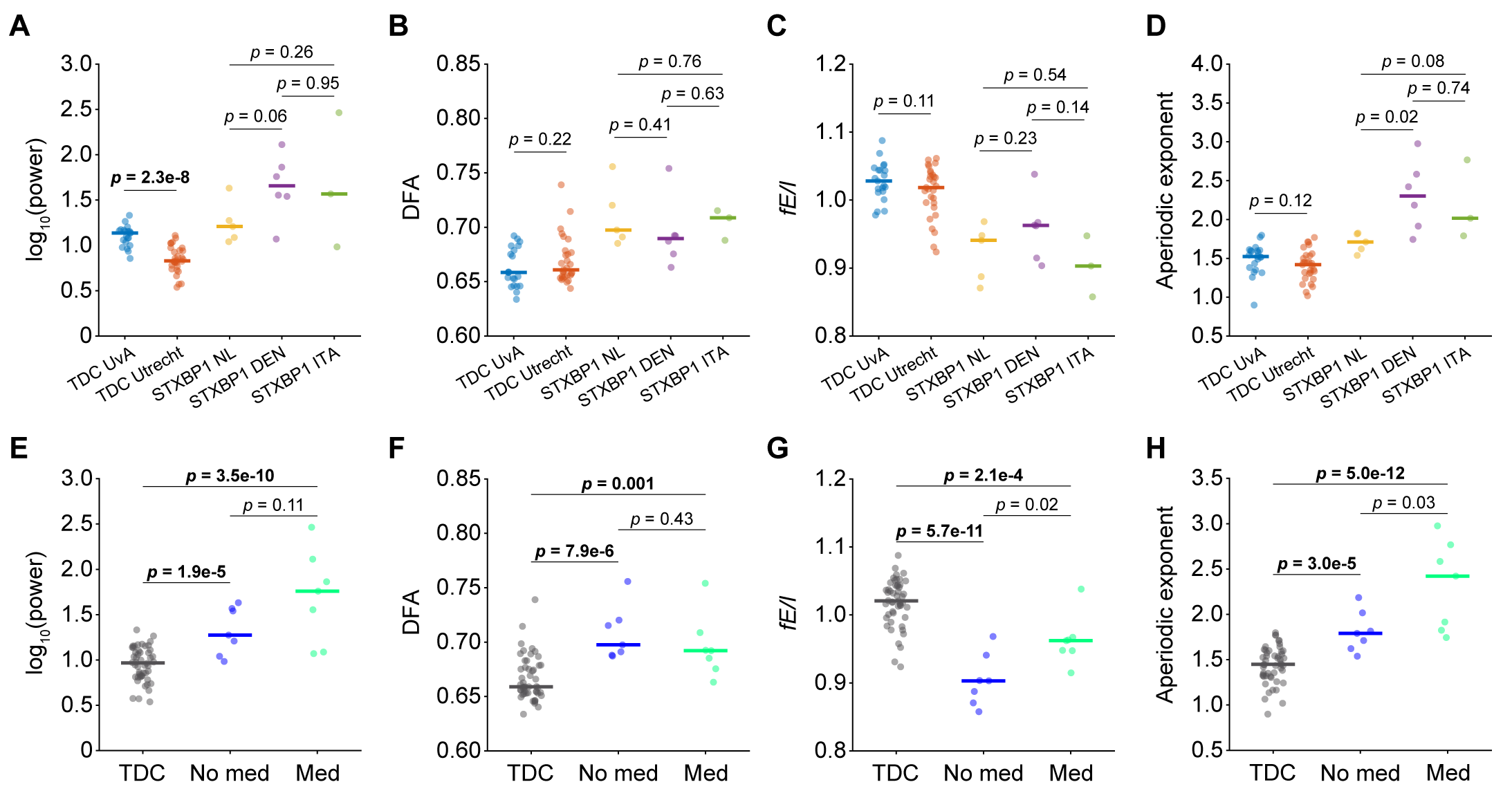

Supplement: Supplementary Figure 5 — Comparisons of sub-cohorts indicate homogeneity of EEG findings across sub-cohorts of the same condition and indicate no difference between patients with or without anti-epileptic medication. Independent samples t-test was used to compare whole-brain averaged EEG measures across sub-cohorts. Significance was defined as p<.05, Bonferroni-corrected for the number of comparisons within each panel (i.e., p<.054). (A) Spectral power in the range 1.75–4.63 Hz was significantly different between the two TDC cohorts, but not between any of the three STXBP1 syndrome cohorts. (B–D) There were no significant differences between the two TDC cohorts or between any of the three STXBP1 syndrome cohorts after Bonferroni correction for DFA in the range 11–18 Hz (B), fE/I in the range 12–24 Hz (C) or aperiodic exponent fitted in the range 1–30 Hz (D). (E–H) Since anti-epileptic medication is thought to reduce excitation, we assessed whether EEG measures from STXBP1 syndrome patients with or without medication were significantly different from each other. EEG measures were significantly different from TDC for STXBP1 syndrome patients with or without medication, but not significantly different between the two groups split for medication status. No med: No medication; Med: Medication at the time of recording. [file Image_5.TIF]

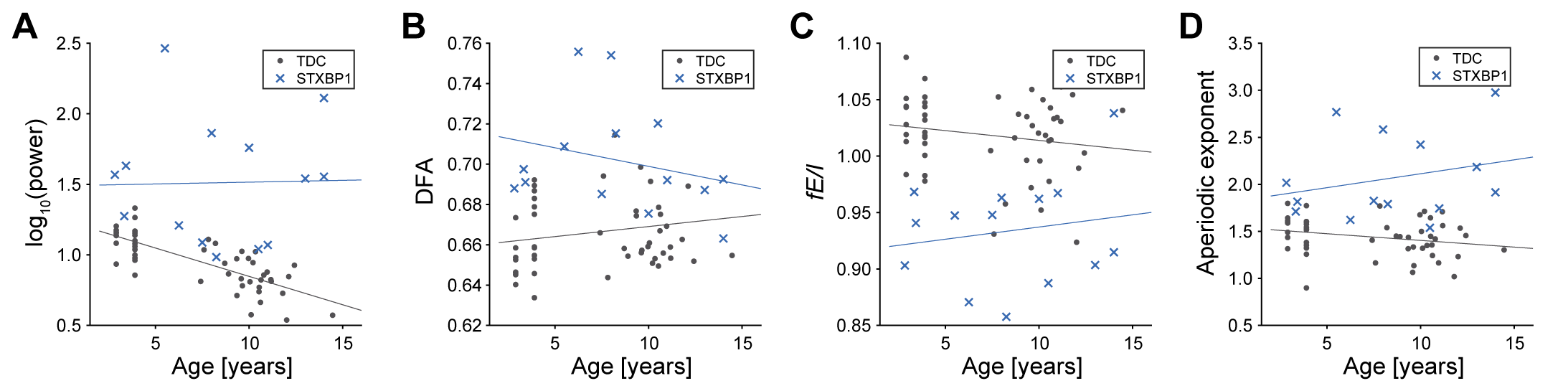

Supplement: Supplementary Figure 6 — Developmental trajectories of EEG measures show altered development of low-frequency power in STXBP1 syndrome. Linear regression was used to fit a model with the whole-brain average of each EEG metric as dependent variable and age as a predictor. Developmental trajectories for TDC (grey) and STXBP1 syndrome (blue) for (A) Low-frequency power, (B) DFA, (C) fE/I, and (D) Aperiodic exponent. [file Image_6.TIF]
